# The Role of Oral Yeasts in the Development and Progression of Oral Squamous Cell Carcinoma: A Scoping Review

**DOI:** 10.3390/jof11040260

**Published:** 2025-03-27

**Authors:** Satutya Wicaksono, Zilefac Brian Ngokwe, Michael McCullough, Tami Yap

**Affiliations:** Melbourne Dental School, University of Melbourne, Carlton, VIC 3053, Australia; satutyaw@student.unimelb.edu.au (S.W.); zilefac.brianngokwe@student.unimelb.edu.au (Z.B.N.); m.mccullough@unimelb.edu.au (M.M.)

**Keywords:** oral squamous cell carcinoma, oral potentially malignant disorders, oral yeasts, *Candida* spp., oral microbiome, oral medicine

## Abstract

The role of oral yeasts in oral squamous cell carcinoma (OSCC) has gained attention due to evidence linking fungal dysbiosis to carcinogenesis. While *Candida albicans* has been the primary focus, emerging studies highlight the importance of non-*Candida* species yeast genera. This scoping review synthesises the evidence on the role of oral yeasts, including *Candida* spp. and non-*Candida* species, in the development and progression of OSCC. A PRISMA-ScR-guided search was conducted in Medline, Embase, EBM Reviews, and CINAHL. Observational and experimental studies involving humans with OSCC, oral potentially malignant disorders (OPMDs), or oral epithelial dysplasia (OED) were included. This review analysed 75 studies. Research on oral yeast in OSCC has progressed since the 1970s, with advancements in identification techniques—from conventional culture methods to metagenomic sequencing and multi-omics approaches—alongside improved animal and cellular models of OSCC. These methodological advancements have identified notable distinctions in the oral mycobiome between carcinomatous and healthy states. Clinical findings reinforce the hypothesis that oral yeasts, particularly *Candida* spp., actively contribute to the dysplasia–carcinoma sequence. Emerging evidence suggests that oral yeasts may significantly modulate events contributing to OSCC progression. However, further mechanistic studies and robust clinical evidence are essential to establish causality and clarify their role in OSCC.

## 1. Introduction

The vital role of the microbiome in cancer development has become increasingly evident. Dysbiotic microbiomes, characterised by shifts in composition and function toward an unhealthy state, have been observed in numerous instances of carcinoma. Evidence suggests that microbial dysbiosis may not only result from disordered tissue architecture but could also precede it [1]. In the context of oral squamous cell carcinoma (OSCC), the role of prominent fungi, such as *Candida* spp., in oral epithelial oncogenesis has been debated for decades, with discussions originating from as early as the 1970s and likely even earlier. These early investigations provided a foundation for understanding the interplay between fungal colonisation and oral epithelial abnormal transformation [2,3,4,5,6,7].

Emerging evidence has highlighted that *Candida* spp., particularly *Candida albicans*, is highly prevalent in patients with oral potentially malignant disorders (OPMDs) and OSCC [8,9]. Their colonisation has been associated with more severe dysplastic changes [10,11,12]. Furthermore, hyphal invasion rates were observed to be higher in tissues exhibiting pronounced oncogenic changes [10,13]. These findings spurred research into the mechanisms by which *Candida* spp. may contribute to oncogenic transformation. Experimental studies have revealed that metabolic by-products, proteolytic enzymes, and other virulence factors of *Candida* spp. could promote events relevant to cancer development and progression [14,15,16]. Despite this, the role of fungi in oral carcinogenesis remains controversial, with ongoing debates regarding the strength of evidence, the precise mechanisms involved, and the lack of robust clinical evidence on the significance of fungal involvement in oral carcinogenesis [17,18].

Advancements in metagenomic and metaproteomic technologies have propelled further exploration of this topic, allowing for a more comprehensive investigation of microbial communities and their functional roles in oncogenesis [19]. Tools like high-throughput sequencing and protein identification software have enabled researchers to uncover not only *Candida* spp. but also other genera, such as *Aspergillus* spp. and *Malassezia* spp., which are increasingly being implicated in OSCC progression. The diversity of fungal genera was observed to be different in OSCC compared to normal tissue or benign lesions, suggesting that various other genera may contribute to the progression of OSCC. Such findings underscore the need to explore beyond *Candida* spp., as these diverse genera might act either synergistically or independently in promoting carcinogenesis [20,21,22,23].

The diverse study approaches used to investigate the role of oral yeasts in oral epithelial carcinogenesis underscore the need for a comprehensive review to synthesise existing evidence on both *Candida* spp. and non-*Candida* yeasts in this context. This scoping review was conducted to assess the breadth of available evidence, examine variations in study methodologies and findings, and identify research gaps to guide future investigations. Specifically, the review aimed to describe the following:

1. The techniques and methodologies used to investigate oral yeast colonisation and/or infection in the context of oral epithelial carcinogenesis.
2. The role of *Candida* spp. colonisation and/or infection in oral epithelial carcinogenesis.
3. The role of non-*Candida* yeast species colonisation and/or infection in oral epithelial carcinogenesis.

## 2. Materials and Methods

We followed the established PRISMA-ScR guidelines in developing the predefined protocol and the final review report [24].

### 2.1. Protocol and Registration

The protocol for this scoping review has been registered with the Open Science Framework (OSF) and is accessible at https://osf.io/m893q (accessed on 25 March 2025).

### 2.2. Eligibility Criteria

The research question was developed using the Population–Concept–Context (PCC) framework. The population included individuals diagnosed with OSCC, oral epithelial dysplasia (OED), or OPMDs, as well as experimental models of these conditions. The concept focused on the role of oral yeasts, and the context explored their impact on OSCC development and progression. The resulting research question was as follows: “What is the available evidence on the role of oral yeasts (*Candida* spp. and non-*Candida* spp.) in the development and progression of oral squamous cell carcinoma?”

Eligible studies included observational, experimental, or clinical research in English involving humans or models of OSCC, OED, or OPMDs, examining the presence, role, or involvement of oral yeasts, particularly their prevalence, pathogenic mechanisms, or interactions with the host or microbiota.

### 2.3. Selection of Sources of Evidence

The search strategy for this scoping review, conducted on 15 August 2024, targeted relevant studies across databases including Medline (via Ovid), Embase (via Ovid), EBM Reviews, and CINAHL. Keyword strings, tailored for each database, combined controlled vocabulary and free-text terms related to oral yeasts (e.g., *Candida* spp.), OSCC, OED, and OPMD, using Boolean operators, truncation, and filters. The strategy, developed with a professional librarian, ensured specificity and sensitivity (keyword strings detailed in Supplementary Materials). Records were de-duplicated using Zotero (v7.0.15.) and uploaded to Covidence^®^.

Screening and selection were conducted by two reviewers. Titles and abstracts were screened against eligibility criteria, with relevant articles undergoing a full-text review. Disagreements were resolved through discussion or consultation with a third reviewer. These processes were conducted in Covidence^®^.

### 2.4. Data Items and Data Charting Process

The data charting process was conducted in Covidence^®^ to systematically capture relevant information aligned with the study’s objectives. Charting forms were calibrated for clarity and consistency, and two reviewers independently performed data extraction, resolving discrepancies through discussion or consultation with a third reviewer. The extracted data included study design, objectives, methods, and statistical analyses related to oral yeasts’ role in oral epithelial carcinogenesis.

### 2.5. Synthesis of Results

The synthesis of results involved systematically organising and summarising the charted data to align with the scoping review’s objectives. The extracted data were collated in a standardised spreadsheet and categorised by study design, objective, method of yeast investigation, and outcome. Bibliometric data were separately organised for analysis.

Descriptive summaries, including the frequencies and distributions of the study characteristics (e.g., study types, geographic locations, and sample collection methods), were tabulated and visually presented. The key findings on yeast prevalence, pathogenic mechanisms, and host–microbiota interactions were summarised narratively. Inferential statistics from the included studies were synthesised to highlight significant associations.

## 3. Results

**Figure 1:**
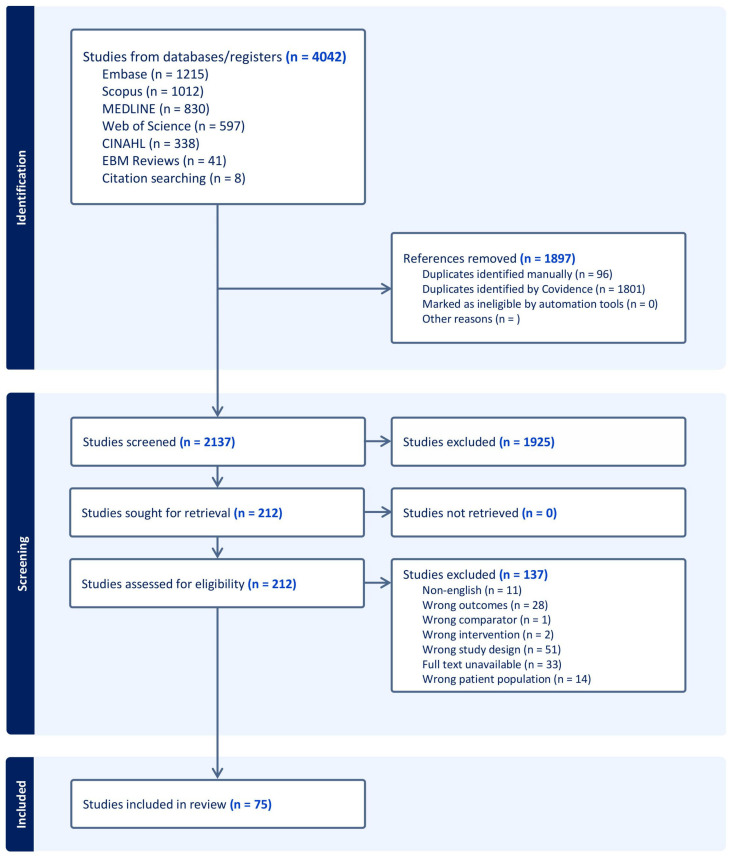
PRISMA-ScR search flowchart.

From the initial pool of 4042 records, 75 articles [2,3,4,5,6,7,8,9,10,11,12,13,14,16,20,21,22,23,25,26,27,28,29,30,31,32,33,34,35,36,37,38,39,40,41,42,43,44,45,46,47,48,49,50,51,52,53,54,55,56,57,58,59,60,61,62,63,64,65,66,67,68,69,70,71,72,73,74,75,76,77,78,79,80,81] met the inclusion criteria and were included in the qualitative synthesis (Figure 1). The included studies comprised a mix of observational and experimental studies, detailed in Tables S1–S3. Observational studies dominated, accounting for 61 out of the 75 included articles.

### 3.1. Study Characteristics

**Figure 2:**
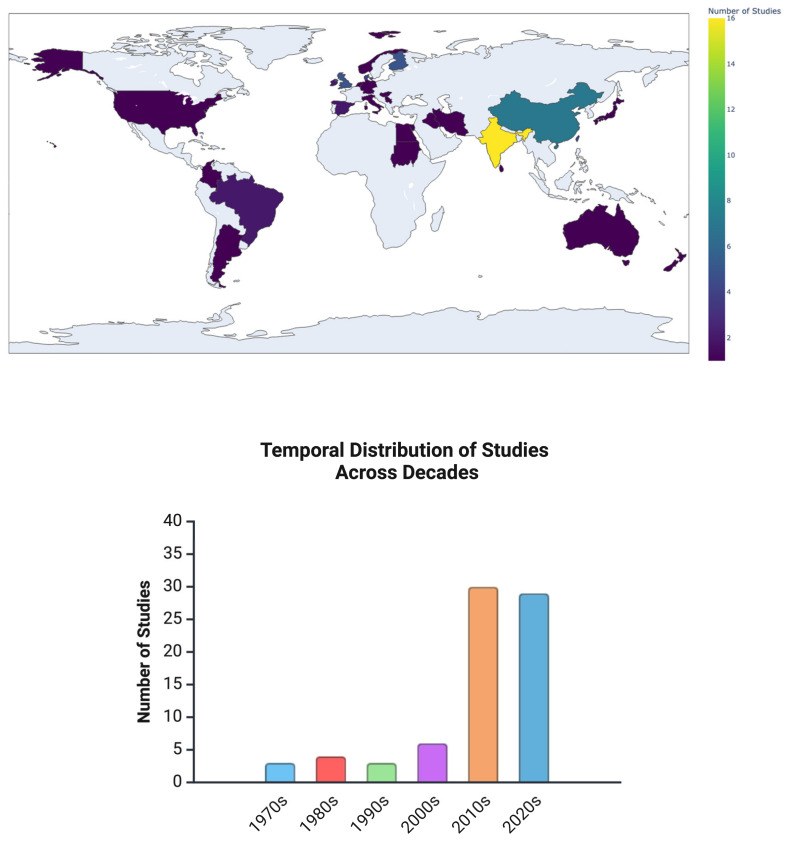
Geographical and temporal distribution of studies on oral yeast in OSCC. The map (**top**) illustrates the geographical distribution of studies, highlighting India as the leading contributor, followed by other regions such as Europe, North America, South America, and the Asia–Pacific. The graph (**bottom**) depicts the temporal distribution of studies, showing a gradual increase in publications from the 1970s to the 2020s, with a significant rise in recent years.

Figure 2 illustrates the geographical and temporal trends in the included studies. Most observational studies were cross-sectional (*n* = 41), followed by case–control (*n* = 12) and cohort (*n* = 9). Additionally, experimental studies included both in vitro (*n* = 9) and in vivo (*n* = 8) models.

The publication dates ranged from 1970 to 2024, with studies originating from diverse regions. While older studies provided historical insights into the role of *Candida* spp. in oral disease progression, recent research has increasingly focused on molecular mechanisms, host interactions, and mycobiome diversity using advanced sequencing and proteomic approaches.

### 3.2. Objective 1: The Techniques and Methodologies Used to Investigate Oral Yeast Colonisation and/or Infection in the Context of Oral Epithelial Carcinogenesis

**Figure 3:**
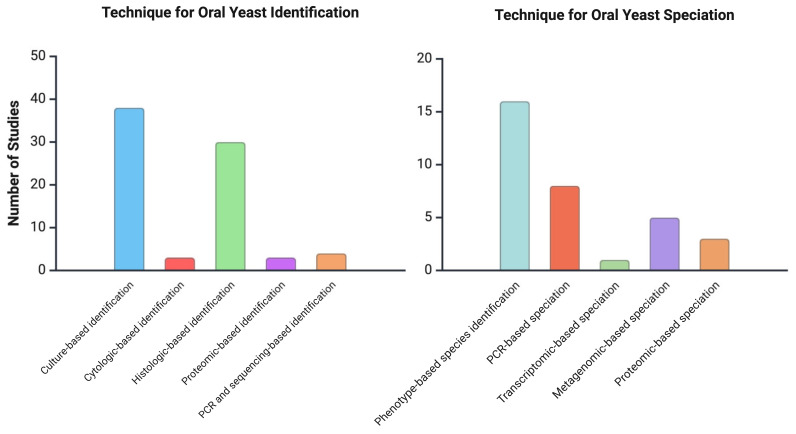
Techniques used for oral yeast identification (**left**) and speciation (**right**) across studies. Culture- and histologic-based methods dominate identification, while phenotype-based and PCR-based approaches are most common for speciation.

The study of oral yeasts in oral epithelial carcinogenesis has evolved significantly over the past few decades, transitioning from traditional culture-based methods to advanced molecular and functional analyses (Figure 3). These methodologies aim to characterise fungal diversity, quantify fungal load, examine their pathogenic potentials, and assess functional interactions between fungi and host tissues.

#### 3.2.1. Culture-Based and Phenotype-Based Identification Techniques

Historically, the identification of oral yeasts relied on culture-based techniques, which remain widely used for fungal isolation and characterisation. Standard culture methods involve plating oral samples on agar-based media, particularly on SDA. Microscopic techniques such as PAS staining and GMS staining have been employed in histological samples to visualise fungal structures in OSCC tissues [22].

For phenotype-based species identification, CHROMagar is commonly used, followed by colony morphology examination and biochemical tests such as germ tube formation, sugar assimilation, and fermentation assays [8,9,32,62]. While these approaches allow for the differentiation of *Candida* spp., they have limitations in detecting mixed fungal communities and low-abundance species.

In clinical settings, automated systems such as Vitek^®^ 2 and Phoenix™ are widely used for yeast identification. These platforms integrate biochemical profiling with advanced algorithms to rapidly and accurately identify fungal species, offering standardisation and efficiency in hospital laboratories. However, such automated methods are generally limited to clinical diagnostics and are rarely used in research applications, particularly in the context of this scoping review. Among the included studies, only Saxena et al. (2021) [48] reported the use of the Vitek^®^ 2 system for yeast identification.

#### 3.2.2. Molecular and Genotypic Characterisation

Advances in molecular biology techniques have enabled more precise identification of fungal species and strains. PCR-based methods have been adopted for detecting and differentiating *Candida* spp. For example, two studies employed PCR-Restriction Fragment Length Polymorphism (PCR-RFLP) and species-specific PCR to identify and distinguish NAC species, such as *C. tropicalis*, *C. glabrata*, and *C. parapsilosis* [8,55]. Moreover, Alnuaimi et al. (2015) [44] described a novel technique called PCR high-resolution melting curve analysis to discriminate species of *Candida* spp. These techniques offer higher sensitivity and specificity compared to phenotype-based approaches. However, the use of PCR-based characterisation of oral yeasts, especially *Candida* spp. is mainly limited to research purposes and is not commonly used in routine clinical diagnostics.

#### 3.2.3. High-Throughput Sequencing and Metagenomic Analyses

With the advent of next-generation sequencing (NGS) technologies, researchers have been able to characterise the oral mycobiome with an unprecedented level of detail. Internal Transcribed Spacer (ITS) sequencing, particularly ITS2-based metagenomics, has become the gold standard for profiling fungal communities in OSCC tissues and saliva samples [21].

High-throughput sequencing studies have revealed significant dysbiosis in the oral mycobiome of OSCC patients, with a reduction in fungal diversity and an enrichment of specific species such as *C. albicans*, *C. etchellsii*, *Malassezia restricta*, *Aspergillus tamarii*, and *Cyberlindnera* spp. [22,23]. These findings suggest that certain fungal taxa may play a role in cancer progression or immune modulation.

Additionally, shotgun metagenomic sequencing has been used to achieve the strain-level resolution of fungal populations, allowing researchers to study genetic variations that may contribute to virulence and acetaldehyde production. Studies using metatranscriptomics (RNA-seq) have provided further insights into fungal metabolic activity and gene expression profiles, helping to elucidate functional interactions between fungi and host cells [79].

#### 3.2.4. Fungal–Bacterial Interaction Studies

Emerging evidence suggests that fungi interact with bacterial communities in the oral cavity, potentially influencing cancer progression. Several studies have utilised both metagenomic and metatranscriptomic approaches to elucidate these interactions. Metagenomic studies, as described by [78,80,81], have employed parallel sequencing of 16S rRNA (bacterial) and ITS (fungal) regions to profile the oral microbiome and assess inter-kingdom microbial relationships. Meanwhile, an integrative metatranscriptomic approach, as employed by Jain et al. (2023) [79], simultaneously characterised both microbial and host transcriptomes. This approach aimed to uncover the mechanisms by which the microbiome (both fungi and bacteria) contributes to oral epithelial carcinogenesis.

#### 3.2.5. Proteomic and Metabolomic Approaches

In addition to genetic and microbiome analyses, proteomic and metabolomic techniques have been employed to investigate fungal contributions to carcinogenesis. Mass spectrometry-based proteomics has been used to compare fungal protein expression between tumour and adjacent non-tumour tissues, identifying fungal proteins associated with immune modulation, oxidative stress, and tissue invasion [20].

Metabolomic studies have also focused on acetaldehyde production, a key carcinogenic metabolite generated by *Candida* spp. Using gas chromatography–mass spectrometry (GC-MS), researchers have quantified acetaldehyde levels in saliva and tissue samples, demonstrating that *C. albicans* can produce mutagenic concentrations of acetaldehyde in the presence of ethanol [16,57,58].

#### 3.2.6. *In Vitro* and *In Vivo* Experimental Models

Functional studies have been conducted using both in vitro and in vivo models to assess the tumour-promoting effects of fungi, focusing solely on *Candida* spp.

- *In Vitro* Studies: Oral squamous cell carcinoma cell lines (e.g., SCC25, SCC15, CAL27) and a dysplastic cell model (i.e., DOK) have been co-cultured with *Candida* spp. to examine their effects on cell proliferation, migration, cytokine secretion, and oncogenic signalling [14,36,56,69,73,74,76,77]. Additionally, co-culture models have been developed to study the effects of Candidal biofilm or combined fungal–bacterial biofilm on OSCC cell lines and oral epithelial cells [56,76].
- *In Vivo* Studies: OSCC models (e.g., 4NQO-induced and xenografted) have been used to assess the impact of fungal colonisation on tumour development. Studies have shown that *C. albicans*-colonised animals develop more severe dysplasia and tumours, accompanied by increased immune suppression and PD-L1 expression. These findings indicate that *Candida* spp. may contribute to immune evasion and tumour progression.

### 3.3. Objective 2: The Role of *Candida* spp. Colonisation and/or Infection in Oral Epithelial Carcinogenesis

#### 3.3.1. Prevalence of *Candida* spp. in OSCC and OPMDs and Its Clinical Implications

The presence of *Candida* spp. in OSCC and OPMDs has been consistently documented across multiple observational studies (Table S1), with a significantly higher prevalence in malignant tissues than in non-malignant mucosa. This suggests a strong association between fungal colonisation and disease progression. McCullough et al. (2002) [10] detected *Candida* spp. in 74.7% of patients with OED or OSCC, compared to 34.2% of those without dysplasia, with higher fungal loads observed in moderate-to-severe dysplasia cases. Similarly, Alnuaimi et al. (2015) [44] identified *Candida* spp. in 69.2% of OSCC cases, compared to 42.3% of controls, with *C. albicans* being the dominant species.

#### 3.3.2. Clinical Impact of Candida spp. Colonisation

The clinical impact of *Candida* spp. colonisation on pre-cancerous and cancerous conditions was described in several studies. A higher fungal burden has been associated with an increased risk of recurrence in premalignant lesions. Chiu et al. (2011) [13] reported that MOLs with *Candida* spp. infection had a significantly higher recurrence rate (67.7%) compared to non-infected MOLs (32.3%) (OR = 4.2, *p* < 0.05). In another study, Mohamed et al. (2021) [21] observed that a higher Candida spp. burden was correlated with poorer overall survival in OSCC patients (*p* = 0.043), suggesting its potential as an independent prognostic factor. Additionally, research by Bansal et al. (2018) [46] highlighted that *Candida* spp. was present in 88.6% of OSCC cases and 45.7% of pre-cancerous lesions, and was completely absent in healthy individuals. Further supporting this, Alnuaimi et al. (2015) [44] found that the presence of *Candida* spp. was identified as a significant risk factor for OSCC (OR = 3.242), with high colonisation further increasing the risk (OR = 3.587).

#### 3.3.3. Prevalence of Non-albicans Candida spp. (NAC) Species in OSCC and OPMDs

While *C. albicans* is the most frequently detected species, several studies have highlighted the presence of NAC species in OSCC and OPMDs (Table S1), suggesting a broader role for fungal diversity in disease progression. Sankari and Mahalakshmi (2019) [9] found that *C. albicans* and NAC species were equally prevalent (41.26%) in OSCC patients, whereas *C. albicans* was more common in healthy controls (55.9%), indicating a greater role of NAC species in malignancy.

Among NAC species, Bansal et al. (2018) [46] reported that *C. krusei* (19.6%) and *C. tropicalis* (9.7%) were the most frequently detected NAC species in OSCC. Similarly, Makinen et al. (2018) [47] found that although *C. albicans* predominated (84%) in OSCC, other NAC species, including *C. dubliniensis*, *C. tropicalis*, and *C. glabrata*, were also present.

Notably, Sankari et al. (2020) [8] observed that NAC species dominated across OSCC and OPMD samples, with *C. krusei* (21%), *C. tropicalis* (21%), and *P. anomala* (21%) being the most prevalent in OSCC. The greater biofilm-forming capabilities of NAC species, compared to *C. albicans*, may contribute to persistent colonisation, enhanced resistance to antifungal treatments, and tumour-promoting effects. These findings suggest that NAC species play a significant role in oral carcinogenesis and warrant further investigation into their pathogenic potential [8].

#### 3.3.4. Mechanisms of *Candida* spp. in Oral Epithelial Carcinogenesis

Several mechanisms have been proposed to explain the oncogenic potential of *Candida* spp., including acetaldehyde production, virulence factor expression, biofilm formation, and interactions with bacterial communities.

#### 3.3.5. Acetaldehyde Production and Carcinogenicity

One of the most well-documented mechanisms is the ability of *C. albicans* to metabolise ethanol into acetaldehyde, a recognised carcinogen (Table S2). Gainza-Cirauqui et al. (2013) demonstrated that *C. albicans* isolates from potentially malignant oral lesions produced acetaldehyde above the carcinogenic threshold (>100 µM) in the presence of ethanol, particularly in smokers [57]. This finding aligns with research by Bakri et al. (2014), who identified elevated ADH1 gene expression in *C. albicans* isolates from chronic hyperplastic candidosis (CHC) lesions, reinforcing its role in localised acetaldehyde production [30].

Further supporting these findings, Marttila et al. (2013) reported that *C. albicans* isolates from OSCC patients produced significantly higher acetaldehyde levels under low-oxygen conditions compared to isolates from controls and APECED patients. Notably, acetaldehyde production was correlated with increased expression of *ALD6* and *ACS1*, genes involved in the pyruvate bypass pathway, while *ADH1* and *ADH2* levels were also upregulated in OSCC-derived strains [58]. These findings suggest that metabolic adaptations in *C. albicans* within the tumour microenvironment may enhance its carcinogenic potential. Additionally, a case–control study assessing *Candida* spp. isolates from 52 OSCC patients and 104 matched healthy controls found that a higher percentage of isolates from OSCC patients produced higher mutagenic acetaldehyde levels (>40 µM) compared to the controls [16].

#### 3.3.6. Virulence Factors and Tissue Invasion

In addition to acetaldehyde metabolism, *C. albicans* employs several virulence factors that may contribute to tissue invasion, immune modulation, and tumour progression. Proteolytic enzyme secretion, particularly aspartyl proteinases and phospholipases, has been implicated in epithelial barrier disruption and inflammatory modulation (Table S2). Rehani et al. (2011) reported that aspartyl proteinase activity was significantly elevated in OSCC tissues compared to non-cancerous controls, supporting its role in tissue degradation [59].

Furthermore, biofilm formation has been shown to enhance fungal persistence and resistance to host immune responses. Alnuaimi et al. (2016) observed that *Candida* spp. isolates from OSCC exhibited increased biofilm formation, phospholipase production, and metabolic activity, which may facilitate the persistent colonisation and local inflammatory responses that drive carcinogenesis [16].

Further evidence of *C. albicans* virulence in precancerous and dysplastic tissues was provided by Krögh et al. (1987), who found that lesion-derived *C. albicans* strains exhibited higher nitrosation potential, converting N-benzylmethylamine into carcinogenic NBMA. Additionally, these strains were more efficient in nitrate-to-nitrite reduction, further facilitating nitrosamine formation, a process implicated in malignant transformation [51].

#### 3.3.7. Immunomodulatory Properties

Beyond its ability to invade tissues and alter metabolic pathways, multiple studies confirm the involvement of *Candida* spp. in shaping the tumour immune microenvironment and in promoting immune evasion and chronic inflammation, which may promote OSCC progression (Table S2). By interacting with immune receptors, cytokines, and tumour-associated immune cells, *Candida* spp. suppresses anti-tumour immunity while inducing immunosuppressive conditions conducive to cancer development.

*Candida* spp. colonisation has been linked to immune dysregulation in OSCC tissues, particularly through the Toll-like receptor (TLR) and NF-κB pathways. Rusanen et al. (2024) found that OSCC tissues exhibited reduced expression of TLR1–5, TLR7, and TLR8 in the basement membrane and endothelium, while infiltrative tumour zones showed increased TLR1, TLR2, TLR4, TLR8, and TLR9 expression, suggesting localised immune activation. Notably, the presence of *Candida* spp. was correlated with higher TLR4 expression in the basement membrane (*p* = 0.012), linking fungal colonisation to inflammatory signalling. Additionally, NF-κB activation was elevated in OSCC infiltrative zones, correlating with increased TLR9 and TLR10 expression, reinforcing its role in chronic inflammation and tumour progression [65].

Another study described the capacity of *Candida* spp. in recruiting immunosuppressive myeloid cells, facilitating tumour immune escape. Wang et al. (2023) demonstrated that in a 4NQO-induced OSCC model, *Candida* spp. infection increased tumour incidence and progression, along with higher tumour-associated macrophages (TAMs), particularly M2-like macrophages, which suppress T cell-mediated immunity. This effect was driven by IL-17A signalling, which upregulated CCL2, a chemokine that recruits macrophages. Neutralising IL-17A reduced macrophage infiltration and tumour progression, confirming the role of *Candida* spp. in immune suppression [71].

Further supporting this, Wang et al. (2024) found that *Candida* spp. infection led to an increase in IL-17A+ CD4+ T cells and γδ T cells, both involved in chronic inflammation and tumour progression. Additionally, *Candida* spp. colonisation expanded polymorphonuclear myeloid-derived suppressor cells (PMN-MDSCs), which actively suppress T cell activation. Blocking CCL2 signalling significantly reduced MDSC infiltration and restored anti-tumour immunity [72].

Beyond shaping the tumour microenvironment, *Candida* spp. has been implicated in upregulating programmed death-ligand 1 (PD-L1), a key immune checkpoint molecule that inhibits T cell-mediated tumour clearance. Wang et al. (2022) showed that *Candida* spp. infection significantly increased PD-L1 expression in OSCC cells through the TLR2/MyD88 and NF-κB pathways, linking fungal infection to chronic-inflammation-driven immune suppression. Notably, in vivo studies confirmed that PD-L1 expression was highest in OSCC tissues colonised by *Candida* spp., indicating its role in tumour immune escape [73].

In addition to local immune modulation, *Candida* spp. infection has been linked to systemic immune suppression, particularly in aged and immunocompromised individuals. Bhaskaran et al. (2021) found that aged mice infected with *Candida* spp. developed OSCC more rapidly, with higher Treg:CD8 ratios, suppressing anti-tumour immunity; increased MDSC infiltration, further dampening T cell activity; and elevated IL-1β levels, promoting chronic inflammation [70]. Similarly, Lee et al. (2020) demonstrated that *Candida* spp., when co-cultured with *Fusobacterium nucleatum*, enhanced IL-1β secretion, activating the PI3K/Akt pathway and worsening tumour-promoting inflammation [36].

Moreover, emerging evidence suggests that *Candida* spp. may also impair the efficacy of immune checkpoint inhibitors (ICIs). Wang et al. (2024) showed that *Candida* spp. infection weakened PD-1 blockade therapy, leading to T cell exhaustion and reduced tumour control, highlighting fungal colonisation as a potential factor in immunotherapy resistance [72].

#### 3.3.8. Fungal–Bacterial Interactions in OSCC Progression

Microbiome profiling of OSCC tissues by Mukherjee et al. (2017) revealed significant microbial dysbiosis, with reduced fungal diversity and strong inter-kingdom correlations. *Candida albicans* and *Lichtheimia* spp. were positively associated with *Fusobacterium* spp. and *Porphyromonas* spp., suggesting cooperative interactions that may drive inflammation and tumour progression [78]. Similarly, Jain et al. (2023) identified transcriptionally active fungi, particularly *Malassezia restricta*, which interacted with pro-tumorigenic bacteria and modulated host pathways linked to proliferation, immune evasion, and carcinogenesis. Their findings further demonstrated that fungal–bacterial consortia were associated with the upregulation of proliferation-related pathways in host cells [79].

Experimental models have further demonstrated how fungal–bacterial interactions enhance tumour progression. Arzmi et al. (2019) showed that polymicrobial biofilms containing *C. albicans*, *Streptococcus mutans*, and *Actinomyces naeslundii* increased oral cancer cell proliferation and migration, accompanied by the upregulation of IL-6 and MMP1, key mediators of tumour invasion [56]. Lee et al. (2020) further found that co-culturing *C. albicans* with *Fusobacterium nucleatum* led to increased IL-1β secretion via the PI3K/Akt/GSK-3β pathway, a mechanism implicated in chronic inflammation and immune suppression [36].

Beyond individual interactions, Heng et al. (2022) reported that *C. tropicalis* was enriched in both OPMDs and OSCC, frequently coexisting with dysbiotic bacterial species linked to biofilm formation and inflammation. This suggests that fungal–bacterial consortia may sustain tumour-supportive conditions, promote persistent infections, and alter immune responses, reinforcing OSCC progression [81].

### 3.4. Objective 3: The Role of Non-*Candida* Yeast Species Colonisation and/or Infection in Oral Epithelial Carcinogenesis

While *Candida* spp. has been the primary focus of research on fungal involvement in OSCC, increasing evidence suggests that other yeast species may also play a role in carcinogenesis (a list of studies with this scope are presented in Table S3). Advances in metagenomic sequencing and fungal proteomics have uncovered a more complex fungal community within OSCC tissues, shifting the perspective from a single-species model to one involving broader fungal dysbiosis. This paradigm shift suggests that interactions between different fungal taxa and the host may contribute to tumour progression through diverse mechanisms.

Recent studies have highlighted key non-*Candida* spp. yeast species that appear to be enriched in OSCC tissues compared to non-malignant counterparts. Perera et al. (2017) demonstrated that while fungal diversity in OSCC tissues was generally lower, carcinogenic fungi such as *Candida etchellsii*- and *Hannaella luteola*-like species were overrepresented. Conversely, fibro-epithelial polyps—considered non-malignant—exhibited an abundance of *Malassezia restricta*, *Aspergillus tamarii*, and *Alternaria alternata*. These findings indicate that while some fungal species might promote tumour progression, others could serve as commensals or even exert protective effects [22]. Further supporting this notion, Mohamed et al. (2021) found that higher *Malassezia* carriage was associated with improved survival outcomes in OSCC patients, suggesting a potentially complex, context-dependent role of certain fungal species [21].

Beyond simply being present in OSCC tissues, several yeast species appear to exhibit oncogenic potential through metabolic and immune-modulatory pathways. Sankari et al. (2020) reported that *C. krusei*, *C. tropicalis*, and *P. anomala* were significantly more prevalent in OSCC than in healthy controls, with *C. famata* exclusively detected in OSCC and OPMDs. The absence of *C. famata* in healthy individuals suggests that it may contribute to malignant transformation rather than merely existing as an opportunistic coloniser [8]. Jain et al. (2023) further demonstrated that transcriptionally active *Malassezia restricta* influenced host cell signalling pathways involved in inflammation and immune evasion, reinforcing the idea that non-*Candida* spp. fungi can modulate the tumour microenvironment [79].

One of the most intriguing aspects of non-*Candida* spp. yeast involvement in OSCC is their interaction with bacterial communities. Mukherjee et al. (2017) observed that *Lichtheimia* spp. and *Malassezia* spp. Were correlated with pathogenic bacterial genera such as *Fusobacterium* spp. and *Porphyromonas* spp., both of which are implicated in OSCC progression. These findings suggest that fungal species may not act in isolation, but rather, contribute to a broader microbial dysbiosis that influences tumorigenesis [78]. Sami et al. (2023) also reported that *Aspergillus* spp., a known aflatoxin producer, was enriched in OSCC samples from Toombak users—a population known for its high oral cancer risk—while *Candida* spp. were more prevalent in non-users. This raises questions about how different fungal taxa interact with external carcinogenic exposures such as smokeless tobacco [80].

Proteomic analyses provide additional insights into the potential functional roles of non-*Candida* yeast species in OSCC. He et al. (2023) identified fungal proteins enriched in OSCC tissues that were associated with tissue invasion, oxidative stress, and immune modulation. *Lichtheimia corymbifera*, *Aspergillus fumigatus*, and *Verruconis gallopava* were particularly abundant in OSCC samples, and their metabolic profiles suggested potential contributions to carcinogenic processes [20]. Similarly, Jain et al. (2023) found that non-*Candida* spp. yeasts interacted with host immune pathways, potentially driving inflammation and tumour progression [79].

These findings collectively underscore the need to reassess the role of fungal communities in OSCC. While *Candida* spp. (especially, *C. albicans*) remains the most well-characterised fungal species in oral cancer, the presence and activity of other yeast species suggest a more nuanced landscape in which multiple fungal taxa, in concert with bacteria and host factors, contribute to disease progression.

## 4. Discussion

This scoping review synthesises the current evidence on oral yeasts in OSCC, revealing a transition from prevalence-focused studies to mechanistic investigations exploring fungal virulence and interactions within the tumour microenvironment. The data consistently indicate that *C. albicans* is the most frequently detected species in OPMDs, OEDs, and OSCC, with its prevalence increasing in tandem with disease severity [10,11,12,43]. However, recent studies have drawn attention to the role of NAC species and other fungal taxa, challenging the conventional view that *C. albicans* is the primary fungal agent implicated in OSCC development [8,20,21,22,23,60,79,80,81].

The elevated detection of *Candida* spp. in dysplastic and OSCC lesions compared to healthy controls has been widely documented, with some studies reporting a three- to four-fold higher prevalence in OSCC patients. While *C. albicans* remains dominant, NAC species, particularly *C. tropicalis*, *C. glabrata*, and *C. parapsilosis*, have been frequently identified in precancerous and malignant lesions [8,46,47,48,49,50]. Beyond *Candida* spp., other fungal genera have also been detected in different compositions in OSCC tissues, including *Rhodotorula* spp., *Saccharomyces* spp., *Kloeckera* spp., *Malassezia* spp., and *Lichtheimia* spp. These findings indicate that shifts in fungal species composition, rather than the presence of *C. albicans* alone, may contribute to OSCC pathogenesis. Differences in fungal diversity between OSCC and healthy oral mucosa have also been observed, with some studies reporting reduced fungal diversity in OSCC, while others indicate a distinct fungal profile with functionally relevant species alterations. This observation implies that certain fungal communities may be more adapted to tumour microenvironments, potentially facilitating carcinogenesis. However, the involvement of non-*Candida* spp. genera in OSCC has only been described in observational studies, and their carcinogenic potential remains unexplored in experimental settings [20,22,23,78,81].

A central debate in this field concerns whether oral yeasts actively contribute to carcinogenesis or merely colonise dysregulated epithelial environments opportunistically. Several studies support an active pathogenic role for oral yeasts, particularly *Candida* spp., highlighting their production of carcinogenic metabolites like acetaldehyde [44,57,58] and N-nitrosobenzylmethylamine [51,82], as well as fungal invasion observed histopathologically in dysplastic and OSCC tissues. Virulence factors including fungal proteases, phospholipases, and immune evasion strategies further suggest their involvement in immune modulation, epithelial degradation, and tissue invasion [11,35,36,59,72,73]. Other virulence factors, including biofilm formation, aspartyl proteases, and phospholipases, enhance immune evasion, epithelial degradation, and tissue invasion, further supporting the notion of a pathogenic role for *Candida* spp. in OSCC [16,55,56,59,60]. Recent studies have expanded this discussion beyond *Candida* spp., identifying other yeasts such as *Malassezia* spp., *Rhodotorula* spp., *Lichtheimia* spp., *Aspergillus* spp., and *Saccharomyces* spp., which potentially contribute through chronic inflammation, cell cycle disruption, oxidative stress, and epithelial damage [20,79,80,81]. Additionally, fungal–bacterial interactions have emerged as significant, with *Candida* spp. frequently co-occurring alongside pro-inflammatory bacteria like *Fusobacterium nucleatum* and *Streptococcus mutans*, suggesting a possible synergistic effect in OSCC pathogenesis. These findings highlight the complexity of fungal involvement in OSCC, underscoring the need for longitudinal and experimental studies to clarify the exact nature of fungal contributions to carcinogenesis [36,56,78,79,81].

Conversely, an alternative perspective suggests that fungal colonisation in OSCC may be a secondary consequence of tumour-induced immune suppression and epithelial barrier breakdown rather than a primary driver of carcinogenesis. The frequent detection of *C. albicans* and other yeasts in non-dysplastic conditions, such as OLK and OLP, indicates that fungal presence alone might be insufficient to initiate malignant transformation. However, the current evidence remains divided, underscoring the necessity for longitudinal studies and experimental models to clarify whether the role of fungi in OSCC is causal or merely opportunistic [15,83,84].

While most studies agree that *Candida* spp. prevalence increases with disease severity, the extent to which fungi actively contribute to carcinogenesis remains contested. *C. albicans*, in particular, has been frequently associated with OSCC, producing carcinogenic metabolites such as acetaldehyde and nitrosamines, and exhibiting increased hyphal invasion in dysplastic and malignant tissues compared to non-dysplastic oral mucosa [10,16,35,44,54]. However, emerging evidence underscores the need to move beyond a *Candida* species-centric perspective and consider the broader oral mycobiome in carcinogenesis. There is ongoing debate about fungal diversity’s role in OSCC, with some studies noting reduced diversity, potentially favouring high-virulence *Candida* spp. strains, while others suggest that diversity changes may be methodological artefacts. Recent evidence highlights that specific fungal communities, including NAC species, could modulate metabolic pathways, host immune responses, and microbial interactions within the tumour microenvironment. Furthermore, the observed increased prevalence of NAC species in OSCC could reflect a shift in microbial ecology rather than direct oncogenic activity [84,85]. Clarifying the exact contribution of fungi, including the broader oral mycobiome, remains essential and underscores the importance of moving beyond a *Candida* species-centric view. The potential impact of antifungal treatments on OSCC risk is another critical area requiring further investigation through longitudinal and experimental studies [17].

### Limitations of This Review

Although this scoping review comprehensively maps the available evidence, several limitations should be acknowledged. A key limitation is the absence of a formal risk-of-bias assessment, which is not typically conducted in scoping reviews. However, potential biases such as publication bias, selective reporting, and methodological inconsistencies across studies may influence the findings. For instance, studies with positive associations between *Candida* spp. and OSCC may be more likely to be published, while studies with null findings might be underreported. Additionally, variations in study design, sample size, and diagnostic methods could introduce selection bias, measurement bias, or confounding factors that affect the interpretation of results.

Furthermore, methodological inconsistencies across the included studies limit the direct comparability of findings. Variability in fungal detection methods, including differences in sampling techniques, culture conditions, and molecular identification approaches, has resulted in significant discrepancies in prevalence rates. The reliance on cross-sectional and in vitro studies restricts the ability to establish causality, while heterogeneity in patient populations, including differences in age, lifestyle factors such as smoking and alcohol consumption, and geographic variability, complicates direct comparisons. Additionally, inconsistencies in histopathological classification criteria for dysplasia and OSCC further affect study comparability, making it difficult to draw definitive conclusions. The inclusion of both observational and experimental studies introduces further heterogeneity, as differences in study design may influence the strength and reliability of the evidence base.

## 5. Conclusions and Implications

This review highlights the complexity of oral yeast involvement in OSCC and the ongoing debate over whether these fungi are active carcinogenic agents or opportunistic colonisers. The growing recognition that fungal diversity in OSCC differs from that in healthy individuals suggests a potential functional role in tumour development, particularly through alterations in microbial interactions, metabolic activity, and immune modulation. However, whether these fungal changes are a driver or consequence of carcinogenesis remains uncertain. Addressing these knowledge gaps through well-designed longitudinal studies, standardised methodologies, and advanced multi-omics analyses will be essential in refining our understanding of fungal contributions to OSCC and identifying potential diagnostic and therapeutic strategies.

## Data Availability

No new data were created or analyzed in this study.

## Supplementary Materials

The following supporting information can be downloaded at: https://www.mdpi.com/article/10.3390/jof11040260/s1, Table S1: Prevalence and characterisation of *Candida* spp. in oral mucosal lesions and OSCC; Table S2: Mechanistic findings of *Candida* spp. and oral carcinogenesis; Table S3: Findings from mycobiome studies on oral squamous cell carcinoma; Table S4: Keywords strings (Medline by Ovid); Table S5: Keywords strings (Embase by Ovid); Table S6: Keywords strings (EBM Reviews); Table S7: Keywords strings (Web of Science).

## Abbreviations

The following abbreviations are used in this manuscript:

CFU	Colony-Forming Unit
DNA	Deoxyribonucleic Acid
EBM	Evidence-Based Medicine
EGFR	Epidermal Growth Factor Receptor
EMT	Epithelial–Mesenchymal Transition
FFPE	Formalin-Fixed Paraffin-Embedded
GMS	Gomori Methenamine Silver
HNSCC	Head and Neck Squamous Cell Carcinoma
HRP	Horseradish Peroxidase
IHC	Immunohistochemistry
IL-6	Interleukin-6
JAK-STAT	Janus Kinase-Signal Transducer and Activator of Transcription
KEGG	Kyoto Encyclopedia of Genes and Genomes
LFC	Logarithmic Fold Change
MMP	Matrix Metalloproteinase
mRNA	Messenger Ribonucleic Acid
NAC	Non-albicans *Candida*
NF-κB	Nuclear Factor Kappa B
OED	Oral epithelial dysplasia
OPMD	Oral potentially malignant disorder
OSCC	Oral squamous cell carcinoma
OTSCC	Oral tongue squamous cell carcinoma
PAS	Periodic Acid–Schiff
PCR	Polymerase Chain Reaction
PRISMA-ScR	Preferred Reporting Items for Systematic Reviews and Meta-Analyses for Scoping Reviews
qPCR	Quantitative Polymerase Chain Reaction
RPKM	Reads Per Kilobase of Exon Model per Million Mapped Reads
RT-PCR	Real-Time Polymerase Chain Reaction
SDA	Sabouraud’s Dextrose Agar
scSeq	Single-Cell Sequencing
TLR	Toll-like receptor
TNF-α	Tumour Necrosis Factor Alpha
TGF-β	Transforming Growth Factor Beta
URM	Upstream Regulatory Mechanism
Wnt	Wingless-Related Integration Site (Wnt Signalling Pathway)
