# Supplementary material for: The Role of Oral Yeasts in the Development and Progression of Oral Squamous Cell Carcinoma: A Scoping Review
Source: J Fungi (Basel). 2025 Mar 27;11(4):260. doi: 10.3390/jof11040260 (PMC12028735; doi:10.3390/jof11040260)
Supplement: Supplementary file 1 [file jof-11-00260-s001.zip › jof-3505877-supplementary.pdf]

Table S1. Prevalence & Characterisation of Candida spp. in Oral Mucosal Lesions and OSCC.

Author and Location	Study Design	Population	*Candida* spp. Identification and Characterisation	Main Findings
**A. Prevalence studies**				
Renstrup, 1970 [2] - Denmark	Cross-sectional	Archival biopies from 235 patients with OLK (i.e., speckled and homogeneous types)	PAS tissue staining	*Candida* spp. was detected in 23% of all OLKs, with higher prevalence in speckled (61%) than homogeneous types (3%). Epithelial atypia was found in 71% of speckled OLKs (61% with *Candida* spp. and 88% without) but absent in homogeneous lesions. Histologically, *Candida* spp. hyphae were present in the superficial epithelium, associated with hyperparakeratosis, inflammatory infiltration, and keratin layer separation.
Roed-petersen [3] et al., 1970 - Denmark	Cross-sectional	- OLK (98 biopsies; 191 smears) Clinically normal oral mucosa (31 biopsies; 197 smears).	PAS tissue staining on tissue & mucosal scrapes samples.	*Candida* spp. was detected in 31% of OLK biopsies but absent in normal mucosa, with 95% agreement between smears and biopsies. *Candida* spp. invasion was more common in OLKs with atypia (67%) than those without (23%), though hyphal density did not correlate with atypia presence.
Daftary et al., 1972 [4]- India	Cohort	723 OLK	PAS tissue staining and culture-based *Candida* spp. isolation	Atypia was associated with candidal infection in seven (14.3%) non-homogenous OL cases (two ulcerated and five speckled). No atypia was detected in homogenous OL.
Hornstein et al., 1979 [5] - Germany	Cross-sectional	- 207 Leukoplakic lesion<br>- 137 Healthy controls	Solid fungal culture (Grütz-Kimmig's and rice agar plates) & PAS tissue staining.	The presence of fungi-positive biopsies was observed to be the highest in squamous carcinomas (35.7%); precancerous OLK (21.1%). Meanwhile, fungi-positive oral smears was found in OSCC; precancerous OLK; benign nosigenous OLKs.
Silverman Jr et al., 1984 [6]- USA	Cohort	257 OLK patients	PAS tissue staining and culture-based *Candida* spp. isolation	31% of patients were *Candida* species-positive. Fungal presence was not related to malignant transformation, occurring in only 2 of 45 patients who developed carcinomas (2.5%)
Barrett et al., 1998 [25] – United Kingdom	Cross-sectional	- 200 squamous papilloma<br>- 138 moderate OED<br>- 238 severe OED	PAS-tissue staining.	Fungal infection was detected in 18% of moderate and 15.2% of severe dysplasia cases, compared to 10% in squamous papilloma. Significantly associated with moderate-severe dysplasia, with infected lesions 3× more likely to progress histologically.

Author and Location	Study Design	Population	*Candida* spp. Identification and Characterisation	Main Findings
McCullough et al., 2002 [10] – United Kingdom	Case-control	- 62 OEDs (20 mild, 17 moderate, 16 severe)<br>- 15 OSCC<br>41 non-OED	Fungal load (CFU/mL) and PAS tissue staining	- ***Candida*** **spp. was** found in **74.7% of OED/OSCC cases vs. 34.2% of non-dysplastic lesions** (p < 0.001).<br>- **High yeast levels (>1000 cfu/mL)** in **83.3% of moderate/severe dysplasia or OSCC** vs. **58.6% in mild dysplasia** (p = 0.017).<br>- PAS staining detected hyphae in **19 patients, 84.2% of whom had high yeast levels** (p = 0.029).
Vučković et al., 2004[26] - Serbia	Cohort	12 OLK	PAS tissue staining and culture-based *Candida* spp. isolation	*Candida* spp. was present in 9/30 cases (30%), including 3/12 (25%) OLK cases, 4/9 (44.4%) OLP, and 1/1 squamous papilloma. The presence of *Candida* spp. was associated with epithelial dysplasia.
Chiu et al., 2011 [13] - Taiwan	Retrospective cohort	- 31 MOLs smoker.<br>- 105 SOLs smoker.	PAS tissue staining.	- *Candida* spp. was more prevalent in MOLs (47.9%) than SOLs (19%) (OR = 3.9).<br>- Dysplasia was higher in *Candida* species-infected MOLs (28.6%) vs. non-infected MOLs (7.9%) but not significant in SOLs.<br>- *Candida* species-infected MOLs had higher recurrence rates (67.7%) than non-infected MOLs (32.3%) (OR = 4.2).<br>- Heavy smokers with MOLs had more *Candida* spp. infections (70.6%) than moderate smokers (28.2%) (OR = 6.11).<br>- *Candida* species-infected MOLs had shorter disease-free time per Kaplan-Meier analysis.
Dany et al., 2011 [27]- India	Case-control	30 OLK and matched controls	Cytological methods, PAS staining	*Candida* spp. was detected in 37% of OLK cases, increasing with lesion stage (14% in stage 1, 42% in stage 2, 66% in stage 4). Dysplasia was more frequent in *Candida* species-positive lesions (55% moderate, 27% mild, 18% no dysplasia), suggesting *Candida* spp. is more common in advanced OLK and dysplasia.
Odedra et al., 2013 [28] - India	Cross-sectional	30 OLK cases (26 non-homogenous, 4 homogenous), 10 OLP, 10 OSMF.	Culture-based (SDA), quantification of CFU, and PAS tissue staining.	*Candida* spp. was isolated in **76% of patients**, with higher prevalence in **moderate/severe OED (88%)** vs. **mild OED (53%).** Colonisation **>1000 CFU/mL** was more common in **moderate/severe OED (79%)** than in mild cases (22%).<br>**Hyphal invasion** was absent in **mild OED** but detected in **32% of moderate** and **73% of severe OED cases**, strongly correlating with **high *Candida*** spp**. levels** (>1000 CFU/mL).
Wu et al., 2013 [29] - China	Retrospective cohort	- 337 general OLK cases<br>- 59 candidal OLK cases	PAS tissue staining.	Candidal OLK was found in **15.9% of patients (59/396)**, primarily on the **tongue (66.1%)** and **buccal mucosa (20.3%). Dysplasia was more common in candidal OLK (55.9%)** than general OLK **(33.5%, p = 0.001). Risk factors** included **age ≥60 (OR 2.28), tongue lesions (OR 1.89), and dysplasia (OR 2.02).**

Author and Location	Study Design	Population	*Candida* spp. Identification and Characterisation	Main Findings
Hebbar et al., 2013 [12] - India	Cross-sectional	- 21 OPMDs without OED.<br>- 14 OPMDs with mild OED.<br>- 6 OPMDs with moderate OED.<br>- 9 Grade 1 OSCC.	Species identification (CHROMagar) and PAS tissue staining.	- Higher *Candida* spp. colonisation correlated with dysplasia severity, with positivity rates of 83.3% in moderate, 57.1% in mild, and 23.8% in non-dysplastic cases.<br>- PAS staining detected hyphae in 50% of moderate dysplasia and 55.6% of Grade 1 OSCC (p = 0.015), indicating a role in dysplastic progression.<br>- High *Candida* spp. levels (>1000 CFU/mL) were more frequent in moderate dysplasia (33.3%) and Grade 1 OSCC (44.4%). Non-homogeneous OLK showed the highest fungal growth (87.5%) among lesion types.
Bakri et al., 2014 [30] – New Zealand	Cross-sectional	- 10 CHC<br>- 8 epithelial keratosis (no dysplasia, no *Candida* spp. infection)<br>- 10 epithelial keratosis (moderate/severe dysplasia, no *Candida* spp. infection)<br>- 3 normal oral mucosa.	PAS tissue staining & IHC (*C. albicans*-specific rabbit antibody for *C. albicans* ATCC 10261 cells))	*C. albicans* was present in 100% of CHC lesions (9/9) but absent in non-dysplastic (0/10) and dysplastic leukoplakia (0/10). PAS staining and immunocytochemistry confirmed its presence in CHC, with no invasion beyond the keratin layer.
Singh et al., 2014 [31] - India	Cross-sectional	OSMF (15), homogenous OLK (13); OLP (6), OLR (7), speckled OLK (4), verrucous OLK (2), tobacco pouch keratosis (3)	Cytology (PAS staining) & histopathology (PAS tissue staining)	- *Candida* spp. was detected in **52% of cytosmears** but only **4% of histopathological sections**, with hyphal forms found in **OSMF and lichenoid reactions.** ***Candida*** spp**. prevalence was highest in cytosmears** from **OLP (83.3%)** and **OSMF (66.7%)**, but histopathology showed it only in **mild dysplastic OSMF and non-dysplastic lichenoid reactions**.<br>- **Dysplasia was present in 32% of cases**, with **speckled OLK (100%)** having the highest prevalence, though **mild dysplasia was most common**. No significant correlation was found between *Candida* spp**. presence and dysplasia severity (p = 0.308).**
Sarkar and Rathod, 2014 [32] - India	Cross-sectional	- 40 OLK<br>- 21 healthy controls	Gram staining and cultured on SDA Species Identification (germ tube and	- *Candida* spp. was more prevalent in OL cases (47.5%) than in controls (14.3%) via direct microscopy (p < 0.001) and culture (45% vs. 4.8%, p < 0.001). *C. albicans* was confirmed in 35% of OL cases through germ tube tests.<br>- Non-homogeneous OLK showed higher *Candida* spp. detection than homogeneous OLK via direct microscopy (64.7% vs. 34.8%, p < 0.01) and culture

Author and Location	Study Design	Population	*Candida* spp. Identification and Characterisation	Main Findings
			cornmeal agar), GMS tissue staining.	(64.7% vs. 30.4%, p < 0.001). *Candida* spp. positivity did not significantly differ between non-dysplastic (36.4%) and dysplastic OLK (48.3%) (p > 0.05).<br>- Hyphal invasion was found in 42.5% of OL cases, with no significant difference between lesion types.
Brouns et al., 2014 [33] - Netherland	Prospective cohort	53 OLK cases	PAS tissue staining.	***C. albicans*** was detected in **26% (14/53) of OLK sections.** Among **16 OLK cases that underwent malignant transformation, PAS-D sections were available for eight patients,** with **four testing positive for *C. albicans*** (P = 0.101**).**
Hongal et al., 2015 [34]- India	Cohort	70 patients (29 OLK, 16 OSMF, 25 OLP)	PAS and GMS tissue staining	Significant association between fungal hyphae and epithelial dysplasia (P = 0.003). Prevalence of *Candida* spp. in dysplastic lesions: 44.4% (OLK), 66.7% (OLP), 100% (submucous fibrosis). Higher grades of dysplasia correlated with increased *Candida* spp. presence.
Tamgadge et al., 2017 [11] - India	Cross-sectional	- 10 mild OED<br>- 10 moderate OED<br>- 10 severe OED<br>- 30 OSCC<br>- 20 healthy controls	Calcofluor white (CFW) tissue Staining	- *Candida* spp. prevalence increased with lesion severity, found in 10% of controls, 76.7% of dysplasia cases (mild: 30%, moderate: 80%, severe: 100%), and 96.7% of carcinoma cases. *Candida* spp. was mainly superficial but penetrated deeper in severe dysplasia and carcinoma.<br>- Colony numbers rose with severity, from 1–2 in controls/mild dysplasia to 6 in severe dysplasia/carcinoma, showing a significant correlation with dysplasia severity and carcinoma (p < 0.05).
Hafed et al., 2019 [35] - Egypt	Cross-sectional	- 16 OED cases<br>- 16 OSCC without LNM<br>- 15 OSCC with LNM<br>- 7 Healthy controls	PAS tissue staining.	*Candida* spp. hyphae was identified in 6.25% of severe dysplasia cases, 31.25% of OSCC without LNM, and 33.3% of OSCC with LNM. No *Candida* spp. was detected in controls.
Lee et al., 2020 [36]	Cross-sectional	- 80 OSCC<br>- 80 Adjacent normal tissue	GMS tissue staining and PCR	*C. albicans* was detected in 11.3% of OSCC cases, indicating its presence in the tumour microenvironment and ability to invade oral cells.
Erira et al., 2021 [37] - Colombia	Cross-sectional	- 16 Homogenous OLK<br>- 14 non-homogenous OLK	PCR	- *C. albicans* was detected in 23.3% of samples, with higher prevalence in severe dysplasia (66.6%) compared to moderate (18.1%) and mild dysplasia (7.6%).<br>- Significant association was found between *C. albicans* presence and dysplasia severity in OLK (p < 0.05).

Author and Location	Study Design	Population	*Candida* spp. Identification and Characterisation	Main Findings
Zhang et al., 2021 [38] - China	Retrospective cohort	48 CHC.	Yeast identification (PAS and GMS tissue staining)	In **CHC cases, dysplasia was detected in 20.83%** (mild: 10.42%, moderate: 8.33%, severe: 2.08%**)**, with **4.17% progressing to SCC** within **6.5 months.** Among **28 cultured CHC cases, 85.71% had *C. albicans* alone,** while ***C. glabrata* and *C. tropicalis*** were found in mixed infections.
Yang et al., 2022 [39] - Taiwan	Retrospecitve cohort	84 patients with a history of OSCC	PAS tissue staining	- OLK had a 25% malignant transformation rate, with an annual transformation rate of 5.73% and a mean transformation time of 4.36 years.<br>- *Candida* spp. infection (HR 5.11, $p = 0.018$), lesions located on the tongue or floor of the mouth (HR 1.18, $p = 0.039$), and multifocal lesions (HR 4.56, $p = 0.047$) were all associated with an increased risk of malignant transformation.<br>- Dysplasia, lesion size, and lifestyle factors (smoking, alcohol, betel quid) were not significant in multivariate analysis.
Rezazadeh et al., 2022 [40] - Iran	Cross-sectional	- 40 OLP (with or without dysplasia)<br>- 32 healthy patients	Fungal load (CFU), species identification (germ tube production CHROMagar)	- *C. albicans* was the only species in OLP (100%) and the most common in controls (92%). Colony counts were similar between OLP (mean 26.68) and controls (23.25, $p = 0.3$).<br>- *Candida* spp. prevalence did not differ between dysplastic and non-dysplastic OLP lesions.
Saraneva et al., 2023 [41] - Finland	Cross-sectional	183 OTSCC patients	PAS tissue staining	- Intraepithelial *Candida* spp. hyphae were found in 21.3% of OTSCC patients, primarily in females (64.1%) and older individuals (mean age 66.1 years, $p = 0.003$), and were more common in non-alcohol users ($p = 0.012$).<br>- OLP and LR were significantly associated with females ($p < 0.001$) and lower rates of smoking and alcohol consumption, but showed no significant link to *Candida* spp. hyphae in OTSCC patients.<br>- OLP/LR patients with *Candida* spp. hyphae may be at risk for OTSCC, despite lacking traditional risk factors like smoking or alcohol use.
B. Characterisation of *Candida* spp.: Non-albicans *Candida* spp.				
Saigal et al., 2011 [13] - India	Cross-sectional	- 15 healthy control<br>- 15 OPMDs<br>- 15 OSCC	Culture-based (SDA), Species identification (Germ tube tests and chlamydospore production assessment.	Fungal growth was absent in the control group but detected in **53.3% of OLK, 20% of OSMF, and 66.7% of OSCC cases**. ***C. albicans*** was confirmed in all OLK and OSMF cases, while only **46.7% of OSCC cases** were ***C. albicans*-positive**, with the remainder involving **NAC species. *C. albicans* presence** was **significantly associated** with **potentially malignant and malignant lesions ($p < 0.01$).**
Gall et al., 2013 [43] - Italy	Cross-sectional	- 48 OSCC<br>- 55 precancerous lesion	PAS and GMS tissue staining, species identification (germ	- *Candida spp.* were found in 30% of OSCC patients and 32% of those with precancerous lesions, with *C. albicans* being the most common species (20.4% in cancer, 28.2% in precancer), followed by *C. glabrata* and *C. tropicalis.*

Author and Location	Study Design	Population	*Candida* spp. Identification and Characterisation	Main Findings
			tube test and the API 20C AUX system)	- Rare *Candida* spp. (*C. incospicua*, *C. famata*, *C. kefyr*) and *Saccharomyces cerevisiae* were isolated only from cancerous lesions.
Alnuaimi et al., 2015 [44] - Australia	Case-control	- 52 OSCC cases<br>- 104 matched control	Species identification (CHROMagar, PCR high-resolution melting curve analysis)	- NAC species accounted for **37.3% of isolates in non-oral cancer patients and 20.5% in oral cancer patients.**<br>- ***C. parapsilosis*** **(47.3%)** was significantly more frequent in non-cancer controls (p = 0.016), while ***C. dubliniensis***, ***C. glabrata***, ***C. tropicalis***, ***C. krusei***, and ***C. guilliermondii*** were also detected at lower frequencies.
Castillo et al., 2018 [62] - Argentina	Cross-sectional	-25 OSCC cases<br>-11 atypical OLP<br>-25 chronic candidiasis<br>15 asymptomatic carrier	CHROMagar, colony morphology, germ tube test, and biochemical assays.	- *Candida albicans* (63%) was the most prevalent species, followed by *C. tropicalis*, which was more common in chronic candidiasis.<br>- Chronic Candidiasis: *C. albicans* (44%), *C. tropicalis* (28%), *C. krusei* (20%), *C. glabrata* (8%)<br>- OLP: *C. albicans* (55%), *C. tropicalis* (18%), *C. krusei* (18%), *C. dubliniensis* (9%)<br>- OSCC: *C. albicans* (64%), *C. tropicalis* (8%), *C. krusei* (16%), *C. dubliniensis* (8%), mixed (4%)<br>- Asymptomatic Carriers: Only *C. albicans* detected.
Hulimane et al., 2018 [45] - India	Cross-sectional	- 32 OED<br>- 18 OSCC<br>- 50 normal oral mucosa	Swab samples from lesions were collected, cultured on SDA, and species identification was performed using CHROMagar.	- *Candida* spp. was present in 100% of dysplasia and OSCC cases, compared to 4% in healthy controls (p < 0.001).<br>- NAC species (66%) were more common than *C. albicans* (34%) in lesions, with *C. tropicalis* (38%) and *C. glabrata* (24%) as predominant species.<br>- NAC species were more frequent in dysplasia (78%), while *C. albicans* predominated in OSCC (56%), though not statistically significant (p > 0.001).
Bansal et al., 2018 [46] - India	Cross-sectional	- 35 oral precancer cases<br>- 39 OSCC<br>- 35 healthy controls	Solid culture using SDA and CHROMagar-based species identification.	- *Candida* spp. was detected in 88.6% of OSCC patients, 45.7% of precancer patients, and none of the healthy participants.<br>- *C. albicans* accounted for 80.9% of all cases, comprising 71% of isolates in OSCC patients (followed by 19.6% *C. krusei* and 9.7% *C. tropicalis*) and 100% of isolates in precancer patients.
Makinen et al., 2018 [47] - Finland	Cross-sectional	- 100 OCSCC patients undergoing treatment<br>- 75 age-matched healthy controls.	Species identification (CHROMagar, Latex agglutination test, API ID 32C yeast identification kit)	- 74% of OCSCC patients harbored *Candida* spp., predominantly *C. albicans* (84%). NAC species included *C. dubliniensis* (8%) and others like *C. tropicalis* and *C. glabrata*.<br>- *Candida* spp. prevalence was similar in OCSCC patients (74%) and controls (63%). Higher anaerobic microbial loads and hyposalivation were linked to increased yeast growth.<br>- *Candida* spp. presence was not significantly associated with mortality in OCSCC. Smokers were more likely to harbor NAC species, and Stage IV patients had higher yeast loads, though not statistically significant.

Author and Location	Study Design	Population	*Candida* spp. Identification and Characterisation	Main Findings
Sankari & Mahalakshmi, 2019 [9] - India	Case-control	- 90 OSCC patients<br>- 170 sex-matched healthy subjects	Fungal load (CFU), Species identification(CHROM agar, germ tube tests, chlamydospore formation on cornmeal agar, sugar assimilation, and fermentation tests).	- Oral *Candida* spp. was higher in OSCC patients (70%) compared to controls (20%).<br>- In OSCC, *C. albicans* and NAC species were equally prevalent (41.26%). In controls, *C. albicans* was more common (55.9%).<br>- High *Candida* spp. CFUs (>1000 CFU/mL) were found in 63.5% of OSCC patients, while controls had lower counts (<1000 CFU/mL). Oral *Candida* spp. in OSCC showed a significant odds ratio (9.33) and risk ratio (3.5).
Sankari et al., 2020 [82] - India	Cross-sectional	- 97 OSCC<br>- 200 OPMDs<br>- 200 Healthy controls	Fungal load (CFU), species identification (CHROM agar, germ tube tests, chlamydospore formation on cornmeal agar, sugar assimilation, and fermentation tests, and PCR-RFLP)	**- *Candida* spp. prevalence was higher in OSCC (72.2%) and OPMD (58%) than in controls (20.5%) (p = 0.0001).**<br>**- NAC species predominated,** with ***C. krusei*** **(21%),** ***C. tropicalis*** **(21%), and** ***P. anomala*** **(21%) in OSCC,** while ***P. anomala*** **(33%) and** ***C. krusei*** **(27%) dominated in OPMD.**<br>**- OSCC had an odds ratio of 4.25 (risk ratio: 11.87), and OPMD had an odds ratio of 3.52 (risk ratio: 6.99*). C. famata*** **was strongly associated with OSCC and OPMD but absent in controls.**
Saxena et al., 2021 [48] - India	Cross-sectional	- 42 smokeless tobacco users<br>- 35 OSCC<br>- 73 Healthy controls (no tobacco use or OSCC)	Species identification (HiCrome agar, VITEK 2 system, fermentation and assimilation).	- *C. krusei* (68%) and *C. glabrata* (28%) were most prevalent in OSCC patients, while C. tropicalis (14%) and *C. krusei* (8%) were dominant in tobacco users. Healthy controls showed lower prevalence, primarily *C. albicans* (12%) and *C. tropicalis* (4%).<br>- NAC species were significantly more prevalent in OSCC.
Abidullah et al., 2021 [49] – India	Cross-sectional	- 50 healthy controls.<br>- 50 OPMDs.<br>- 50 OSCC.	Yeast identification (SDA), species identification (CHROM agar)	- Positive isolates of *Candida* spp. were significantly more frequently found in the OSCC group (84%) than in the OPMD (40%) or control (28%) group.<br>- *C. albicans* was the most frequent species in all groups, followed by *C. krusei*. Meanwhile, different species (e.g., *C. glabrata*, *C. tropicalis*) and multispecies *Candida* spp. were only found in the OSCC group.
Ilhan et al., 2023 [50] - Türkiye	Case-control	- 20 benign lesions without dysplasia	Species identification (RT-PCR)	- *C. albicans* was significantly more prevalent in tissues with mild to moderate dysplasia compared to healthy tissues (p = 0.001), carcinoma in situ (p = 0.031), and OSCC (p = 0.000).<br>- *C. tropicalis* was more frequently detected in mild to moderate dysplasia tissues than in healthy tissues (p = 0.004) and carcinoma in situ (p = 0.019).

Author and Location	Study Design	Population	*Candida* spp. Identification and Characterisation	Main Findings
		- 20 Mild/moderate OEDs<br>- 20 Carcinoma in situ<br>- 20 OSCC cases		- The study observed a notable coexistence of *C. albicans* and *C. tropicalis* in dysplastic tissues, suggesting a potential shift from a commensal state to an opportunistic pathogenic role in the early stages of OSCC.
C. Characterisation of *Candida* species*:* Biotyping *Candida albicans*				
Krogh et al., 1987 [7] - Denmark	Cross-sectional	- 17 OLK (homogenous; non-homogenous).<br>- 19 reticular OLP..	Biotyping of *C. albicans* (Phenotypic assay: acid/salt tolerance, proteinase production, drug resistance, and substrate assimilation; three-digit system) and PAS tissue staining.	- Yeasts were more prevalent in OLK (82%) than OLP (47%) and normal mucosa. *C. albicans* dominated (82% in OLK, 83% in OLP). PAS staining detected yeast hyphae in 50% of OLK but not in OLP.<br>- Rare *C. albicans* biotypes were more common in non-homogeneous OLK. Among 18 identified *C. albicans* biotypes, 355 and 177 were the most frequent.
Rindum et al., 1994 [52] - Denmark	Cross-sectional	21 erythematous candidiasis cases<br>32 leukoplakic lesions (homogenous, nodular, and erythroplakia types)	Solid culture (malt agar), Species identification (*Candida* BCG agar), Phenotype-based *C. albicans* biotyping (nine biochemical tests); Histology/Cytology (PAS staining).	- Yeasts were found in 59% of OLK and 100% of erythematous candidosis, with hyphae more frequent in pathological lesions, especially erythematous candidosis (95%).<br>- *C. albicans* dominated, but pathological lesions had greater yeast diversity. Smokers showed higher yeast colonisation and more diverse *C. albicans* strains. Non-homogeneous OLK had more frequent hyphae and dysplasia, aligning with its higher malignancy risk.
Williams et al., 2001 [53] – United Kingdom	Prospective	A 65-year-old patient with chronic hyperplastic candidosis (CHC) was followed over seven years.	- IR-PCR (1251 primer) identified genetic similarity and persistence of *C. albicans* isolates.<br>- RHE model used for tissue invasion,	DNA fingerprinting confirmed *C. albicans* persistence for seven years despite antifungal treatment. The patient's isolate showed deeper (4-cell) invasion in the RHE model, differing from the shallow, uniform invasion of other strains. The progression of CHC to SCC supports *C. albicans'* role in malignancy

Author and Location	Study Design	Population	*Candida* spp. Identification and Characterisation	Main Findings
			with 24-hour *C. albicans* exposure.<br>- H&E and PAS staining.	
Abdulrahim et al., 2013 [54]- Ireland	Case-control	78 OLK patients (31 Candidal OLK, 47 non-Candidal OLK)	Culture, *C. albicans* genotyping (multilocus sequence typing (MLST), ABC genotyping).	- *Candida* spp. was detected in 39.7% of biopsies, with dysplasia in 90.3% of *Candida*-infected cases (12.9% mild, 45.2% moderate, 32.3% severe).<br>- MLST showed no specific *Candida albicans* clade associated with OLK. However, ABC Genotype C was enriched in Candidal OLK (36%) vs. non-Candidal OLK (15.8%) and healthy carriage (8.8%).<br>- Malignant transformation occurred in 6.5% of *Candida* species-infected OLK cases.
Alnuaimi et al., 2015 [44] - Australia	Case-control	- 52 OSCC cases<br>- 104 age-, gender- and denture status-matched OSCC-free subjects	Culture-based (SDA) and ABC Biotyping (PCR high-resolution melting curve analysis).	- *Candida* spp. was more prevalent in OSCC (69.2%) than controls (42.3%), with high colonisation (>567 cfu/mL) more frequent in cancer patients (36.1%) than controls (13.6%).<br>- *C. albicans* dominated both groups, with genotype A more common in OSCC (87%) than controls (46.9%), while genotype B was higher in controls (50%) than OSCC (13%).<br>- *Candida* spp. presence (OR = 3.242) and high colonisation (OR = 3.587) were significant OSCC risk factors, further amplified by alcohol consumption (OR = 4.253). Combined *Candida* spp. presence and alcohol use increased OSCC risk nearly tenfold (OR = 9.288).
Weerasekera et al., 2021 [55] – Sri Lanka	Case-control	- 80 OLK<br>- 18 healthy controls	Species identification (germ tube test and corn meal agar test and PCR-RFLP), *C. albicans* ABC biotyping.	- Genotype A was predominant (60% in OLK patients, 78% in controls), while Genotypes B and C were less frequent with no correlation to demographic or virulence factors.<br>- No significant link was found between patient habits (smoking, alcohol, betel nut chewing) and genotypes.

Table S2. Mechanistic findings into Candida spp. and Oral Carcinogenesis.

Study & Country	Study Design	Intervention/ Assessment	Model/ Population	Main Findings
**A. Biofilm Formation**				
Alnuaimi et al., 2016 [44] - Australia	Case-control	**Biofilm Analysis:** Crystal violet & tetrazolium salt reduction assay.	*Candida* spp. isolates from: 52 OSCC cases and 104 matched healthy controls	Biofilm forming capacity was observed higher in *Candida* spp. isolates from OSCC cases.
Castillo et al., 2018 [62] - Argentina	Cross-sectional	**Biofilm Formation:** XTT reduction assay	-25 OSCC cases<br>-11 atypical OLP<br>-25 chronic candidiasis<br>15 asymptomatic carrier	- *Candida* spp. from oral lesions showed higher biofilm formation than asymptomatic carriers.<br>- NAC species formed stronger biofilms than *C. albicans*, indicating species-specific virulence potential.
Arzmi et al., 2019 [56] - Australia	*In vitro*	Monospecies and polymicrobial biofilm of *Candida albicans* isolate from oral cancer patient (ALC3), *Actinomyces naeslundii* and *Streptococcus mutans*.	SCC25 (WT p53 gene) & SCC15 (mutant p53 gene)	- *C. albicans* formed strong biofilms, while *S. mutans* and *A. naeslundii* produced weaker ones. Polymicrobial biofilms had greater structural complexity and biomass.<br>- Polymicrobial biofilm factors promoted SCC25 cell proliferation and migration but had no effect on SCC15 cells.<br>- Polymicrobial biofilm factors upregulated inflammation and cancer-related genes (*IL-6, MMP1*) in SCC25 cells, with a weaker effect in SCC15 cells.
**B. Nitrosation Potential**				
Krögh et al., 1987 [88] – Denmark	Cross-sectional	- **Nitrosation Potential:** Yeasts incubated with N-benzylmethylamine and sodium nitrite (pH 6.8), with chemical controls.<br>- **Nitrosamine Detection:** NBMA quantified via HPLC, confirmed by GC-MS.<br>- **Nitrate Reduction:** Griess-Ilosvay's reagents.	- *C. albicans* (21 strains, 15 biotypes)<br>- *C. tropicalis* (2 strains)<br>- *C. parapsilosis* (1 strain)<br>- *T. glabrata* (2 strains)<br>Isolates were collected from 12 oral precancerous lesions (OLK, erythroleukoplakia).	- Lesion-derived *C. albicans* converted N-benzylmethylamine to carcinogenic NBMA more efficiently than normal mucosa strains.<br>- Dysplastic lesion strains reduced nitrate to nitrite, promoting nitrosation.<br>- *C. albicans* biotypes varied in nitrosation activity, with dysplastic lesion strains being the most active.
**C. Acetaldehyde Production**				

Study & Country	Study Design	Intervention/ Assessment	Model/ Population	Main Findings
Gainza-cirauqui et al., 2013 [57] - Spain	Cross-sectional	Yeast Identification (colony morphology, CHROMagar, microscopy, (API ID32C), and confirmed with PCR. Acetaldehyde Assay (gas chromatography).	**Isolates from:** OLLs, 16 OLP, 6 OLK, 6 healthy patients.	- *C. albicans* from potentially malignant lesions produced carcinogenic acetaldehyde (>100 μM) with ethanol.<br>- Isolates from smokers, especially alcohol users, had higher acetaldehyde production in ethanol-glucose incubations.<br>- Control isolates (no lesions) produced even more acetaldehyde, indicating *C. albicans*' innate production potential.
Marttila et al., 2013 [58] - Finland	Cross-sectional	**Yeast Identification** (CHROMagar morphology and negative Bichro-Dubli latex test).<br>**Acetaldehyde & Ethanol Production** (gas chromatography).<br>**Northern blotting** (*ADH1, ADH2, PDC11, ALD6, ACS1, ACS2*)<br>**Alcohol Dehydrogenase Activity:** Fluorescence assays measured cytosolic Adh activity with ethanol (0.68–2174 mM).	-5 OSCC patients<br>-5 APECED patients<br>5 healthy controls	- *C. albicans* produced high acetaldehyde under low oxygen, highest in OSCC strains (716.6 μM), followed by control (654 μM) and APECED strains (619.4 μM).<br>- Acetaldehyde levels correlated with *ALD6* and *ACS1* but not *ADH1, ADH2,* or *PDC11*. OSCC strains had higher *ADH1, ADH2,* and *ALD6* expression.
Bakri et al., 2014 [30] – New Zealand	Cross-sectional	**Molecular Analysis** (RT-PCR for Ca18S rRNA; *CaADH1; CaADH2* mRNA).	**Biopsies of 10** CHC, **8** epithelial keratosis (no dysplasia, no *Candida spp.*), **10** epithelial keratosis (moderate/severe dysplasia, no *Candida spp.*), and **3** normal oral mucosa	- High *C. albicans ADH1* mRNA expression correlated significantly with *C. albicans* presence in CHC lesions.<br>- No significant correlation was found between *ADH2* mRNA expression and *C. albicans* presence in CHC lesions.
Alnuaimi et al., 2016 [44] - Australia	Case-control	**Acetaldehyde Production:** Yeast incubated with 11 mM	*Candida* spp. isolates from:<br>- 52 OSCC cases	- A higher number of OSCC isolates generated mutagenic levels of acetaldehyde (>40 μM) compared to non-OSCC isolates.<br>- Univariate analysis linked ethanol-derived acetaldehyde, alcohol use, and *Candida* spp. colonisation to OSCC.

Study & Country	Study Design	Intervention/ Assessment	Model/ Population	Main Findings
		ethanol and was measured using gas chromatography.	- 104 Matched healthy controls	- Multivariate analysis confirmed biofilm activity, phospholipase, and alcohol use as key OSCC contributors.
Hafed et al., 2019 [35] - Egypt	Cross-sectional	- 16 OED cases<br>- 16 OSCC without LNM<br>- 15 OSCC with LNM<br>- 7 Healthy controls	CaADH1 RNA expression (RT-PCR).	CaADH1 mRNA, linked to acetaldehyde production, was found in 100% of OSCC cases without LNM, 80% with LNM, and one oral dysplasia case, but absent in controls.
D. Proteolytic Enzyme				
Rehani et al., 2011 [59] - India	Cross-sectional	- ***Candida*** **spp. Identification:** Cultured on SDA and identified via HiCandida Kit.<br>- **Colony-Forming Capacity** (hemocytometry after incubation in YPD broth and YCB-BSA medium)<br>- **Secreted Aspartyl Proteinase** (spectrophotometer).	- 10 OSCC cases<br>- 10 OLK patients<br>- 10 Smokers<br>- 10 Healthy non-smokers controls	- Highest in OSCC (9.82 μmol/min/mL, 12.10 CFU/mL), followed by OLK (8.05 μmol/min/mL, 11.42 CFU/mL). Both were significantly higher than in smokers and nonsmokers ($p = 0.001$).<br>- Slightly higher Sap and CCC in smokers, but not significant ($p > 0.05$).<br>- The CCC-Sap correlation showed a positive relationship across all groups with statistical significance ($p < 0.05$).
Berkovits et al., 2016 [60] - Hungary	Case-control	Extracellular Lipase Activity (YNB-rhodamine B plates), Proteolytic Activity (YCB-BSA plates).	20 OSCC cases<br>- 40 Healthy oral mucosa controls	Protease (47.14% vs. 37.5%) and lipase (37.86% vs. 32.5%) of *Candida* spp. production did not significantly differ between OSCC and controls.
Alnuaimi et al., 2016 [44] - Australia	Case-control	- **Enzyme Activity: Aspartyl Proteinase** (YCB-BSA test medium)<br>- **Phospholipase** (Egg-yolk agar)<br>- **Esterase** (Tween-80 opacity test).	*Candida* spp. isolates from:<br>- 52 OSCC cases<br>- 104 Matched healthy controls	- *Candida* spp. from OSCC patients showed higher phospholipase levels but no difference in proteinase or esterase.<br>- Phospholipase activity was identified as a key OSCC risk factor in both univariate and multivariate analyses.
Nawaz et al., 2018 [61] - Finland	Cross-sectional	- **Gelatin Zymography:** Gelatin-SDS-PAGE, Coomassie staining, densitometry.<br>- **Fluorometric Assay:** Enzyme activity measured using specific	**Stock *Candida* spp. Strains:** *C. albicans, C. glabrata, C. krusei, C. tropicalis*<br>**Clinical Isolates (OSCC Patients):** *C. albicans*, *C.*	- *C. tropicalis* showed the highest proteolytic activity, followed by *C. krusei* and *C. glabrata*, with clinical isolates surpassing reference strains.<br>- *C. albicans* exhibited negligible proteolytic activity compared to non-*C. albicans* species.<br>- All species degraded fibronectin and claudin 4, crucial for epithelial integrity, with *C. tropicalis* and *C. krusei* showing the greatest capacity.<br>- Clinical isolates displayed higher proteolytic activity, indicating enhanced virulence.

Study & Country	Study Design	Intervention/ Assessment	Model/ Population	Main Findings
		substrates and a plate reader.<br>- **Protein Degradation:** Fibronectin & claudin 4 incubated with yeast, analysed via SDS-PAGE and staining.	*glabrata*, *C. krusei*, *C. tropicalis.*	
Castillo et al., 2018 [62] - Argentina	Cross-sectional	- **Lipolytic Activity:** Rhodamine-B plaque assa<br>- **Proteolytic Activity:** BSA-supplemented agar method<br>- **Cell Surface Hydrophobicity:** Microbial adhesion to hydrocarbons (MATH) test	-25 OSCC cases<br>-11 atypical OLP<br>-25 chronic candidiasis<br>-15 asymptomatic carrier	- *Candida* spp. from oral lesions showed higher lipolytic, proteolytic activity, and surface hydrophobicity than asymptomatic carriers.<br>- More virulent isolates were found in malignant lesions than in potentially malignant ones, linking *Candida* spp. virulence to severity.
Weerasekera et al., 2021 [55] – Sri Lanka	Case-control	Enzymatic activities (phospholipase, proteinase, haemolysin, esterase, coagulase), biofilm formation, epithelial adherence, and phenotypic switching were assessed using agar-based and *in vitro* methods.	- 80 OLK<br>- 18 healthy controls	*Candida* spp. isolates exhibited high virulence, including phospholipase (80%), proteinase (66%), haemolysin and esterase (100%), coagulase (97%), biofilm formation (77%), and adherence to buccal epithelial cells. Phenotypic switching was rare (8.6%).
E. Immune modulation				
Rodriguez et al., 2007 - [63] Spain	Cross-sectional	Immunohistochemistry (Mab C7 staining; Ab developed against C. albicans' cell wall mannoproteins); Western Blot: SDS-PAGE and immunoblotting.	34 early-stage OSCC samples (T1/T2N0M0)	- Mab C7, targeting *Candida albicans* mannoproteins, strongly reacted with OSCC tissues, detecting 40-47 kDa and 70 kDa proteins in 38.2% of tumors.<br>- High reactivity correlated with greater tumor thickness, lower keratinisation, and perineural invasion.<br>- Elevated Mab C7 expression was linked to shorter survival and aggressive tumor behavior, likely due to Nup88 overexpression, suggesting the potential role of *Candida* spp. in OSCC progression.

Study & Country	Study Design	Intervention/ Assessment	Model/ Population	Main Findings
Hsieh et al., 2022 [64] - Taiwan	Cross-sectional	**Gene expression** (**scRNA-seq**); **Gene & Pathway Analysis** (KRAS, NF-κB, TLR); **Immune Profiling** (T cells, macrophages, and neutrophils); **Gene Annotation; IHC Validation** stratifin (SFN).	**Cell Isolates:**<br>- Healthy gingiva (1 sample)<br>- OPMD lesion (1 sample)<br>- OSCC tumour without *C. albicans* (1 sample)<br>- OSCC tumour with *C. albicans* (1 sample)	- *C. albicans* in OSCC upregulated KRAS and E2F targets, while non-infected OSCC activated IL2/STAT5, TNFα/NFκB, and TGFβ pathways.<br>- *C. albicans*-infected OSCC had fewer T cells and more macrophages/neutrophils, promoting immune evasion.<br>- Elevated SFN in *C. albicans*-infected OSCC suggests its potential as a fungal-associated OSCC marker.<br>- Normal mucosa lacked fungi, while OPMD with *C. albicans* showed early immune responses. OSCC with fungal infection exhibited strong immunosuppression and oncogenic activation.
Rusanen et al., 2024 [65] - Finland	Cross-sectional	Yeast Identification (SDA culture); Immunohistochemistry (TLR1-10 and NF-κB staining).	- 30 OSCC patients<br>- 26 Heathy controls	- Reduced TLR1, TLR2, TLR3, TLR4, TLR5, TLR7, and TLR8 in OSCC vs. healthy controls.<br>- TLR3, TLR4, TLR7, and TLR8 were lower in the BM zone; TLR4, TLR7, and TLR8 decreased in the endothelium.<br>- Increased TLR1, TLR2, TLR4, TLR8, and TLR9, with TLR4 notably elevated.<br>- *Candida* spp. colonisation (23% of OSCC cases) correlated with increased TLR4 in the BM zone ($p = 0.012$).<br>- NF-κB was more prominent in healthy controls but showed upregulation in the infiltrative zone of OSCC, indicating its involvement in tumor progression.<br>- NF-κB positively correlated with TLR9 and TLR10 in OSCC's infiltrative zone ($p = 0.04$, $p = 0.002$).
Franklin & Martin, 1986 [66] - United Kingdom	*In vivo*	*C. albicans* NCPF 3091 (serotype A) was applied in the pouch post-surgery.	Male Syrian hamsters, 4-6 weeks old	- *C. albicans* in turpentine-induced hyperplasia caused dysplasia, resembling Candidal OLK.<br>- Sustained *C. albicans* contact via sutured pouches intensified dysplasia.<br>- Discontinuing *C. albicans* exposure led to epithelial normalisation, highlighting its role in maintaining dysplasia.
O'Grady & Reade, 1992 [67] - Australia	*In vivo*	Inoculating *C. albicans* (strain 151).	4NQO +PDD induced carcinogenesis rat model.	- *C. albicans* and 4NQO increased carcinoma rates in rats, with 71% developing tongue or palate cancers by 52 weeks.<br>- *C. albicans* induced hyperplasia with fungal hyphae in neoplastic lesions but did not cause cancer alone.<br>- *C. albicans* exhibited tumour-promoting activity comparable to PDD, a known chemical promoter.

Study & Country	Study Design	Intervention/ Assessment	Model/ Population	Main Findings
Dwivedi et al., 2009 [68] – (USA)	*In vivo*	*C. albicans* suspension (6 × 10^8 yeast/mL)	Immunocompetent C57BL/6 female mice, 3 to 5 weeks old	- *C. albicans* infection post-4NQO exposure induced dysplastic lesions.<br>- Elevated Ki67 and p16 confirmed enhanced proliferation and dysplastic transformation.<br>- *C. albicans* alone caused hyperplasia, while 4NQO or *C. albicans* alone did not induce dysplasia.
Chen et al., 2020 [69] - China	*In vitro*	Zymosan (100 micrograms/mL)	OSCC cell (WSU-HN4, WSU-HN6, & CAL27)	Zymosan enhanced OSCC cell proliferation via TLR2/MyD88, increased *C. albicans* adhesion, and elevated IL-1β levels, promoting a pro-inflammatory environment that may aid cancer progression.
Bhaskaran et al., 2021 [70] - USA	*In Vivo*	Prolonged sublingual inoculation *C. albicans* (SC5314) blastospores or zymosan	4-NQO induced carcinogenesis model (young mice; aged mice; TLR-2-/- and Dectin-1-/- knockout mice.	- Aged 4-NQO-treated mice developed tumours faster, with increased Tregs, MDSCs, and immune suppression.<br>- Dectin-1 promoted OSCC progression; KO mice had lower IL-1β, fewer Tregs, and slower tumour growth.<br>- Treg depletion reduced OSCC severity, restoring PD-1 and IFN-γ in CD8+ T cells, enhancing immunity.<br>- Elevated IL-1β in aged mice and OSCC tissues recruited Tregs and MDSCs, promoting tumour growth.<br>- Aged mice had more fungal DNA; *Candida* spp. or zymosan exposure induced dysplasia, IL-1β, and Treg recruitment.
Wang et al., 2023 [71] - China	*In vivo*	*C. albicans* strain SC5314	- 4NQO-induced oral carcinogenesis model (Male C57BL/6N mice).<br>SCC VII cell-injected tongue tumour model (Male C3H/HeN mice).	- *C. albicans* increased tumour incidence and progression, while fluconazole reduced tumour development.<br>- *C. albicans* enhanced TAM infiltration and M2-like polarization, elevating PD-L1 and GAL-9.<br>- IL-17A upregulated CCL2, attracting macrophages; neutralizing IL-17A reduced infiltration and tumour progression.<br>- *C. albicans* and IL-17A promoted M2-like polarisation, promoting an immunosuppressive tumour environment.
Wang et al., 2024 [72] - China	*In vivo*	*C. albicans* (SC5314)	SCC tongue tumour-bearing mice (6–8-week-old C3H/HeN mice).	- *C. albicans* reduced CD8+ T cells and increased IL-17A+ CD4+ T cells, γδ T cells, and PMN-MDSCs in the TME.<br>- IL-17A recruited and activated MDSCs; its neutralisation partially restored CD8+ T cells and slowed tumour growth.<br>- Upregulated CCL2 and CCL20 attracted MDSCs; CCL2 neutralisation reduced PMN-MDSC infiltration and immunosuppression.<br>- *C. albicans* weakened PD-1 blockade efficacy, increasing T cell exhaustion and worsening tumour control.

Study & Country	Study Design	Intervention/ Assessment	Model/ Population	Main Findings
Wang et al., 2022 [73] - China	*In Vitro*	- *C. albicans* strain ATCC 90028 and SC5314<br>- Heat-inactivated *C. albicans*	OSCC cell lines (Cal27 and HN6)	- *C. albicans* infection increased PDL1 expression in OSCC cell (Cal27, HN6) at mRNA and protein levels, with heat-inactivated *C. albicans* showing a similar effect, but not biofilm metabolites.<br>- PD-L1 upregulation was driven by TLR2/MyD88 and TLR2/NF-κB pathways, with variable involvement of EGFR/MAPK and JAK2/STAT pathways.<br>- *C. albicans* infection elevated PD-L1 in normal and OSCC mucosa, with the highest levels in OSCC mice, suggesting enhanced immune evasion.
	In Vivo	*C. albicans* innoculations.	4NQO-induced carcinogenesis mice models.	- Zymosan enhanced OSCC cell growth via TLR2/MyD88.<br>- Zymosan increased *C. albicans* adhesion, promoting colonisation.<br>- Zymosan elevated IL-1β, promoting a pro-inflammatory environment that may induce cancer progression.
O'Grady & O'Sullivan, 2023 [74] - Ireland	*In vitro*	Heat-inactivated *C. albicans* (HICA)	Ca9-22 cells	- Ethanol induced apoptosis in Ca9-22 cells, enhanced by HICA. HICA increased MMP-2, while ethanol inhibited it via TIMP-2.<br>- Ethanol suppressed and delayed NF-κB activation, weakening the immune response to HICA.<br>- Both promoted migration; together, they increased anchorage-independent growth, suggesting higher tumorigenic potential.<br>- No changes in IL-6, IL-8, or other cytokines; IL-10 was consistently downregulated.
F. Pro-proliferative and Oncogenic Effects				
Nakazawa et al., 2007 [75]- Japan	Case-control	Papanicolaou staining, PAS staining, flow cytometry, immunohistochemistry (p53, Ki-67, COX-2)	175 patients (44 OLK, 49 OLP, 42 inflammatory lesions)	*Candida* spp. presence correlated with higher DNA aneuploidy, proliferative index, and p53/Ki-67/COX-2 expression. OLK with *Candida* spp. had the highest dysplasia-related changes (DI = 2.2 vs. 2.0 in non-*Candida* spp.; PI = 75.3% vs. 65.4%).
Lee et al., 2020 [36] - Taiwan	*In vitro*	- **Co-culture:** Live or heat-killed *C. albicans* (SC5314) and *F. nucleatum*.<br>- **LDOC1 Modulation:** Knockdown and ectopic expression in cell lines.<br>- **Pathway Analysis:** PI3K and Akt inhibitors tested for their role in microbial-induced IL-1β production in LDOC1-deficient cells.	Immortalised normal keratinocytes (CGHNK2) and OSCC cell lines (TW2.6).	- LDOC1 suppressed microbial-induced IL-1β, while its deficiency activated PI3K/Akt, increasing IL-1β and promoting oral cancer.<br>- PI3K/Akt/GSK-3β regulated LDOC1-mediated IL-1β suppression; PI3K/Akt inhibition or active GSK-3β reduced IL-1β in LDOC1-deficient cells.<br>- *C. albicans* alone did not induce OSCC but, with carcinogens, promoted tumour development and dysplasia with prolonged exposure.

Study & Country	Study Design	Intervention/ Assessment	Model/ Population	Main Findings
	In vivo	*C. albicans* SC5314 was applied to oral lesions	4NQO + arecoline induced carcinogenesis mice model.	
Amaya-arbelaez et al., 2021[76] - Brazil	*In vitro*	Soluble factors (SF) from single or dual biofilms of *C. albicans* and *S. aureus* were collected at early (16-hour) and mature (36-hour) stages.	NOK-si, SCC25, & Detroit 562 cancer cells.	- SF from *C. albicans* and dual-species biofilms (16h, 36h) reduced NOK-si cell viability but had no effect on SCC25 and Detroit 562 cells.<br>- In NOK-si cells, *S. aureus* SF upregulated *CDKN1A*, inducing G2/M arrest. In SCC25 and Detroit 562 cells, SF upregulated oncogenes (*hRAS, mTOR*) and survival genes (*Bcl-2, CDKN1A*).<br>- SF induced apoptosis in NOK-si cells (increased sub-G0 phase) but caused minimal cell cycle changes in cancer cells.
Marin-dett et al., 2022 [77] - Brazil	*In vitro*	ECLs from *C. albicans* biofilms, rich in phosphatidylinositol (PL), phosphatidylcholine (PC), and phosphatidylglycerol (PG).	DOKs & SCC25.	- ECLs trigger lipid droplet (LD) formation in DOK SCC25, boosting metabolism and apoptosis resistance.<br>- ECLs sequester camptothecin (CPT) in LDs, reducing its cytotoxicity.<br>- ECLs minimally impact viability but increase G1 and reduce G2/M phase in SCC25 cells.
Vadovics et al., 2022 [89] – United Kingdom	*In vitro*	Live or heat-inactivated *C. albicans* and *C. parapsilosis*	OSCC cell lines (HSC-2 and HO-1-N-1)	- ***C. albicans* promotes OSCC progression** by enhancing **migration, invasion, and extracellular matrix degradation** via **MMP activity** and **EMT induction** (increased vimentin, slug; decreased E-cadherin).<br>- **Metabolic shifts** (increased succinic & aspartic acid) fuel tumour growth.<br>- ***In vivo* models** show **accelerated tumour growth, dysplasia, and malignancy**, with **increased immune infiltration and pro-tumour inflammation**.<br>- ***C. albicans* vs. *C. parapsilosis***: Only ***C. albicans*** induces **OSCC invasiveness, MMP secretion, oncogenic gene expression, and EMT**, whereas *C. parapsilosis* **has minimal impact**.
	In vivo	Chronic oral candidiasis (*C. albicans* inoculation).	Xenograft immunodeficient mice model (HSC-2 cells) & 4NQO mice model.	

Table S3. Findings from Mycobiome Studies on Oral Squamous Cell Carcinoma.

Author and Country	Study Design	Samples	Methods	Main Findings
Berkovits et al., 2016[60] - Hungary	Case-control	60 (20 OSCC, 40 controls)	MALDI-TOF	- **Yeast was detected in 90% of OSCC patients vs. 30% of controls,** with a significantly higher fungal burden (**73.08 CFU/cm² vs. 1.10 CFU/cm²).**<br>- **OSCC patients exhibited greater yeast diversity,** mainly ***Candida*** spp., and harboured ***Rhodotorula*** spp. **(9.1%), *Saccharomyces*** spp. **(9.1%), and *Kloeckera*** spp. **(4.5%),** absent in controls.<br>- **Yeast colonisation was higher on neoplastic epithelium (77.38 CFU/cm²) than adjacent healthy tissue** (**28.58 CFU/cm²).**
Perera et al., 2017[22] - Sri Lanka & Australia	Case-control	25 OSCC; 27 fibro-epithelial polyps	- qPCR<br>- ITS2 sequencing (Ilumina MiSeq)<br>- BLASTN classification (UNITE database)<br>- QIIME & LEfSe for taxonomic analysis	- OSCC had significantly lower **species richness** and **alpha diversity** than FEP (Shannon index: **1.5 ± 0.9 (OSCC) vs. 2.1 ± 1.2 (FEP), p < 0.05**).<br>- *C. albicans* (61.2% vs. 29.6% in FEP), *C. etchellsii*, and a *Hannaella luteola*-like species were enriched in OSCC.<br>- *Malassezia restricta*, *Aspergillus tamarii*, *Alternaria alternata*, and a *Hanseniaspora uvarum*-like species were overrepresented in controls.<br>- Despite lower diversity, OSCC samples had higher relative abundance of potentially carcinogenic fungi.
Banerjee et al., 2017[23] - USA	Cross-sectional	100 OSCC; 20 matched controls; 20 non-matched controls	Pan-pathogen microarray (PathoChip) & Next-generation Sequencing (NGS)	- **OSCC tumors had lower Shannon diversity than controls.**<br>- Enriched fungal species in OSCC: ***Rhodotorula mucilaginosa***, ***Geotrichum candidum***, ***Pneumocystis jirovecii***, ***Fonsecaea pedrosoi***, ***Cladophialophora bantiana***, ***Malassezia restricta***.<br>- Enriched fungal species in Controls: ***Cladosporium cladosporioides***, ***Phialophora verrucosa***.<br>- Microbial genome integration suggest fungi may contribute to OSCC progression and its profile can potentially serve as OSCC biomarkers.
Mukherjee et al., 2017[78] - USA	Case-control	39 OTSCC and matched controls.	- 16sRNA & ITS1 sequencing (Ilumina Miseq)<br>- QIIME for taxonomic analysis	- **Fungal richness** was significantly lower in tumor tissues compared to matched normal tissues (p<0.05).<br>- **Fungal diversity** (Shannon index) was not significantly different between tumor and normal tissues.<br>- The fungal genus ***Lichtheimia*** showed significant positive correlations (p<0.05) with pathogenic bacteria (*Fusobacterium*, *Porphyromonas*, *Campylobacter*) and negative correlations with beneficial bacteria (*Actinomyces*, *Lactobacillus*).
Sankari et al., 2020 [82] - India	Cross-sectional	97 OSCC; 200 OPMDs; 200 healthy controls	- Phenotypic assays: CHROM agar, germ tube test, chlamydospore formation, sugar assimilation/fermentation.	- *Candida* spp. carriage was significantly higher in OSCC (72.2%) and OPMD (58%) compared to healthy controls (20.5%) (p = 0.0001). NAC species predominated, notably *C. krusei*, *C. tropicalis*, and *P. anomala*.<br>- OSCC showed an OR of 4.25 (RR: 11.87), and OPMD an OR of 3.52 (RR: 6.99). *C. famata* strongly associated with OSCC/OPMD, absent in controls.

			- Genotypic assay: PCR-RFLP.	
Mohamed et al., 2021 [90] – Sudan & Norway	Cross-sectional	59 OSCC; 13 healthy controls	- qPCR<br>- ITS2 sequencing (Ilumina MiSeq)<br>- QIIME & DADA2 for taxonomic analysis	- Salivary mycobiome was dominated by *Candida* spp., *Saccharomyces* spp., *Malassezia* spp., *Aspergillus* spp., and *Cyberlindnera* spp. Sixteen genera (e.g., *Lodderomyces* spp., *Alternaria* spp.) were exclusively identified in OSCC patients.<br>- Alpha diversity indices (richness and evenness) were not significantly different between OSCC patients and controls. The overall mycobiome composition did not significantly differ (PERMANOVA: p=0.265, ANOSIM: p=0.056).<br>- Higher *Candida* spp. carriage correlated with poorer overall survival (OS) (Kaplan-Meier Breslow test: p=0.043). Conversely, higher *Malassezia* spp. carriage independently predicted favorable OS (HR=0.383, 95% CI=0.16–0.89, p=0.03).
Jain et al., 2023 [91], USA	Cross-sectional	20 OSCC; 20 adjacent normal tissue	- Laser microdissection and deep metatranscriptomic sequencing (RNA-seq).<br>- Microbial profiling via HUMAnN 3.0 (taxonomic and functional profiling).<br>- Host transcriptome (DESeq2 and Gene Set Enrichment Analysis (GSEA)).	- Fungi were present in low abundance but transcriptionally active in OSCC tissues.<br>- ***Malassezia restricta*** was significantly enriched in OSCC tumor and adjacent tissues compared to healthy controls.<br>- ***Candida albicans*** was rarely detected (only one OSCC sample).<br>- The fungal enzyme profiles (metabolic pathways) were broadly similar across OSCC and controls, indicating functional redundancy despite taxonomic differences.<br>- *Malassezia restricta* potentially interacts with host proliferation-related pathways (e.g., MYC and E2F targets), suggesting a possible role in oral carcinogenesis.
Sami et al., 2023 [80] – Sudan & Ireland	Cross-sectional	43 OSCC (19 Toombak users 26; non-Toombak users); 78 healthy controls (47 Toombak users; 31 non-users)	- qPCR (ITS1-ITS2 regions)<br>- ITS1-ITS2 sequencing (Ilumina MiSeq)<br>- Data processing QIIME, DADA2, UNITE ITS database, Microbiome Analyst, and Calypso software.	- ***Aspergillus*** spp. significantly enriched in saliva of Toombak users (78.93%) vs non-users (21.07%).<br>- ***Candida*** spp. significantly depleted in Toombak users (4.33%) vs non-users (95.67%) (q=4.758e−4).<br>- ***Malassezia restricta*** significantly higher in Toombak users (63.02%) compared to non-users (36.98%; q=0.046).<br>- Other genera enriched in Toombak users: ***Blumeria*** spp. **(61.7%)**, ***Issatchenkia*** spp. **(61.52%)**, ***Saccharomyces*** spp. **(62.11%)**.<br>- Genera decreased in Toombak users: ***Metschnikowia*** spp. **(36.22%)**, **Cladosporium** spp. **(65.07%)**.<br>- Increased *Aspergillus* spp. poses risk due to potential aflatoxin exposure, contributing to carcinogenesis in Toombak users<br>- Shift in fungal species (e.g., *Candida tropicalis* dominance in Toombak users) suggests increased virulence and resistance profiles, potentially affecting OSCC progression.
He et al., 2023 [20] - Australia	Cross-sectional	The study analysed **CPTAC proteomic**	- Mass spectrometry (CPTAC dataset), TMT	- Fungi dominated microbial diversity (228 species vs. 24 bacterial, 8 viral) and were more abundant in OSCC.

		**data** from **mass spectrometry** of tumour and adjacent normal tissues in **HPV-negative HNSCC and OSCC patients.**	labelling, Trans-Proteomic Pipeline (TPP)<br>- Taxonomic classification (UniProt, NCBI), hierarchical clustering (Euclidean), log2 fold-change, differential protein analysis (Benjamini-Hochberg, p<0.05)	- The dominant phyla were *Ascomycota* (46%) and *Basidiomycota* (30%). OSCC tissues had 196 enriched fungal proteins (adjusted p<0.05), with 28 overexpressed, compared to only 2 in normal tissues.<br>- Key OSCC-associated fungi included *Lichtheimia corymbifera* (tissue destruction), *Malassezia sympodialis* (cancer progression), and *Paracoccidioides brasiliensis* (chronic inflammation).<br>- Enriched fungal proteins were linked to tissue invasion, oxidative stress, immune modulation, and biofilm formation, suggesting roles in carcinogenesis.<br>- Fungal protein profiles outperformed bacterial and viral markers in distinguishing OSCC, highlighting their diagnostic potential.<br>- *Candida albicans* was undetected, likely due to antiseptic use during sample collection.
Heng et al., 2022 [81] - China	Cross-sectional	- 30 Healthy controls<br>- 32 OPMDs<br>- 29 OSCC	- qPCR<br>- ITS2 sequencing (Ilumina MiSeq)<br>- QIIME & DADA2 for taxonomic analysis	- *Ascomycota* decreased in OSCC (HC: 65%, OSCC: 55%, p=0.025, FDR=0.133).<br>- OSCC-enriched fungi included *Acremonium e*xuviarum, *Aspergillus fumigatus*, and *Candida tropicalis* (LDA >3), while *Morchella septimelata* was depleted (HC: 17%, OSCC: 5%, p=0.018, FDR=0.118).<br>- *Acremonium exuviarum* showed diagnostic potential (AUC = 0.74).<br>- OSCC-associated fungi were linked to oxidative stress, immune modulation, and biofilm formation.<br>- *Candida* spp. and *Acremonium* spp. correlated positively, while *Morchella* spp. and *Clitopilus* spp. shifted from positive to negative correlations in OSCC.

Table S4. Keywords strings (Medline by OVID).

No	Search Terms	Number of Hits
1	Exp Mouth Neoplasms/	79,146
2	((mouth or oral or tongue or palat* or lingual or buccal or lip or labial or retromolar or gingiva*) adj4 ("squamous cell carcinoma" or neoplasm or cancer* or precancer* or malignan* or tumour or tumor or neoplasia or carcinoma or premalignant or pre?malignant or "potentially malignant lesion" or "potentially malignant disorder" or leukopla* or erythropla* or dysplas* or metasta*)).mp.	64,254
3	1 or 2	106,754
4	Exp Yeast/	220,444
5	Exp Fungus/	448,882
6	Exp Mycobiome/	1,580
7	(*Alternaria* or *Aspergillus* or *Aureobasidium* or *Candida* or *Cladosporium* or *Cryptococcus* or *Dothioraceae* or *Eurotium* or *Fusarium* or *Glomus* or *Saccharomyces* or *Saccharomycetales* or *Teratosphaeria* or *Malassezia* or *Irpex* or *Cytospora* or *Valsa* or *Lenzites* or *Trametes* or *Sporobolomyces* or *Sporidiobolus*).mp.	340,138
8	4 or 5 or 6 or 7	533,880
9	3 and 8	715

Table S5. Keywords strings (Embase by OVID).

No	Search Terms	Number of Hits
1	exp mouth cancer/ or exp mouth carcinoma/	58,359
2	exp oral potentially malignant disorder/	2,636
3	((mouth or oral or tongue or palat* or lingual or buccal or lip or labial or retromolar or gingiva*) adj4 ("squamous cell carcinoma" or neoplasm or cancer* or precancer* or malignan* or tumour or tumor or neoplasia or carcinoma or premalignant or pre?malignant or "potentially malignant lesion" or "potentially malignant disorder" or leukopla* or erythropla* or dysplas* or metasta*)).mp.	109,575
4	1 or 2 or 3	106,754
5	Exp Yeast/	91,594
6	Exp Fungus/	653,645
7	Exp Mycobiome/	2,050
8	(*Alternaria* or *Aspergillus* or *Aureobasidium* or *Candida* or *Cladosporium* or *Cryptococcus* or *Dothioraceae* or *Eurotium* or *Fusarium* or *Glomus* or *Saccharomyces* or *Saccharomycetales* or *Teratosphaeria* or *Malassezia* or *Irpex* or *Cytospora* or *Valsa* or *Lenzites* or *Trametes* or *Sporobolomyces* or *Sporidiobolus*).mp.	405,643
9	5 or 6 or 7 or 8	704,048
10	4 and 9	1,099

Table S6. Keywords strings (EBM Reviews).

No	Search Terms	Number of Hits
1	(yeast* or fungi or fungus or mycobiome or *Alternaria* or *Aspergillus* or *Aureobasidium* or *Candida* or *Cladosporium* or *Cryptococcus* or *Dothioraceae* or *Eurotium* or *Fusarium* or *Glomus* or *Saccharomyces* or *Saccharomycetales* or *Teratosphaeria* or *Malassezia* or *Irpex* or *Cytospora* or *Valsa* or *Lenzites* or *Trametes* or *Sporobolomyces* or *Sporidiobolus*).mp.	7,009
2	((mouth or oral or tongue or palat* or lingual or buccal or lip or labial or retromolar or gingiva*) adj4 ("squamous cell carcinoma" or neoplasm or cancer* or precancer* or malignan* or tumour or or neoplasia or carcinoma or premalignant or pre?malignant or "potentially malignant lesion" or "potentially malignant disorder" or leukopla* or erythropla* or dysplas* or metasta*)).mp.	4,415
3	1 and 2	41

Table S7. Keywords strings (Web of Science).

No	Search Terms	Number of Hits
1	ALL=(yeast* or fungi or fungus or mycobiome or *Alternaria* or *Aspergillus* or *Aureobasidium* or *Candida* or *Cladosporium* or *Cryptococcus* or *Dothioraceae* or *Eurotium* or *Fusarium* or *Glomus* or *Saccharomyces* or *Saccharomycetales* or *Teratosphaeria* or *Malassezia* or *Irpex* or *Cytospora* or *Valsa* or *Lenzites* or *Trametes* or *Sporobolomyces* or *Sporidiobolus*)	889,693
2	TS=((oral NEAR/4 malignan*) or (oral NEAR/4 carcinoma) or (oral NEAR/4 cancer) or (oral NEAR/4 neoplas*) or (oral NEAR/4 dysplas*) or (mouth NEAR/4 malignan*) or (mouth NEAR/4 carcinoma) or (mouth NEAR/4 cancer) or (mouth NEAR/4 neoplas*) or (mouth NEAR/4 dysplas*) or (tongue NEAR/4 malignan*) or (tongue NEAR/4 carcinoma) or (tongue NEAR/4 cancer) or (tongue NEAR/4 neoplas*) or (tongue NEAR/4 dysplas*) or (lip NEAR/4 malignan*) or (lip NEAR/4 carcinoma) or (lip NEAR/4 cancer) or (lip NEAR/4 neoplas*) or (lip NEAR/4 dysplas*)or (buccal NEAR/4 malignan*) or (buccal NEAR/4 carcinoma) or (buccal NEAR/4 cancer) or (buccal NEAR/4 neoplas*) or (buccal NEAR/4 dysplas*) or (gingiva* NEAR/4 malignan*) or (gingiva* NEAR/4 carcinoma) or (gingiva* NEAR/4 cancer) or (gingiva* NEAR/4 neoplas*) or (gingiva NEAR/4 dysplas*) or (labial NEAR/4 malignan*) or (labial NEAR/4 carcinoma) or (labial NEAR/4 cancer) or (labial NEAR/4 neoplas*) or (labial NEAR/4 dysplas*) or (retromolar NEAR/4 malignan*) or (retromolar NEAR/4 carcinoma) or (retromolar NEAR/4 cancer) or (retromolar NEAR/4 neoplas*) or (retromolar NEAR/4 dysplas*) or (palat* NEAR/4 malignan*) or (palat* NEAR/4 carcinoma) or (palat* NEAR/4 cancer) or (palat* NEAR/4 neoplas*) or (palat* NEAR/4 dysplas*) or (palat* NEAR/4 leukopla*) or (mouth NEAR/4 leukopla*) or (buccal NEAR/4 leukopla*) or (labial NEAR/4 leukopla*) or (gingiva* NEAR/4 leukopla*) or (retromolar NEAR/4 leukopla*) or (oral NEAR/4 precancer*) or (buccal NEAR/4 precancer*) or (lip NEAR/4 precancer*) or (labial NEAR/4 precancer*) or (mouth NEAR/4 precancer*) or (palat* NEAR/4 precancer*) or (retromolar NEAR/4 precancer*) or (oral NEAR/4 erythropla*) OR (buccal NEAR/4 erythropla*) or (lip NEAR/4 erythropla*) or (labial NEAR/4 erythropla*) or (mouth NEAR/4 eryhtropla*) or (palat* NEAR/4 erythropla*) or (retromolar NEAR/4 erythropla*) or (oral NEAR/4 metasta*) OR (buccal NEAR/4 metasta*) or (lip NEAR/4 metasta*) or (labial NEAR/4 metasta*) or (mouth NEAR/4 metasta*) or (palat* NEAR/4 metasta*) or (retromolar NEAR/4 metasta*))	66,768
3	1 and 2	599
